# Structural conservation and functional role of TfpY-like proteins in type IV pilus assembly

**DOI:** 10.1128/jb.00343-24

**Published:** 2025-01-16

**Authors:** Ikram Qaderi, Isabelle Chan, Hanjeong Harvey, Lori L. Burrows

**Affiliations:** 1 Department of Biochemistry and Biomedical Sciences, McMaster University 3710 , Hamilton, Canada; 2 Michael G. DeGroote Institute for Infectious Disease Research, McMaster University 536887 , Hamilton, Ontario, Canada; University of California San Francisco, San Francisco, California, USA

**Keywords:** *Pseudomonas aeruginosa*, type IV pili, bacteriophage, accessory proteins

## Abstract

Type IV pili (T4P) are important virulence factors that allow bacteria to adhere to and rapidly colonize their hosts. T4P are primarily composed of major pilins that undergo cycles of extension and retraction and minor pilins that initiate pilus assembly. Bacteriophages use T4P as receptors and exploit pilus dynamics to infect their hosts. Some bacteria encode pilin accessory proteins that post-translationally glycosylate major pilins to evade phage binding. TfpY is an accessory protein of unknown function that is widespread and structurally conserved among T4P-expressing bacteria. Here, we use *Pseudomonas aeruginosa* as a model to characterize the functional role of TfpY and its homologues in pilus assembly. TfpY expression is required for optimal pilus assembly and function; however, it does not provide phage defence, unlike previously characterized accessory proteins. TfpY can cross-complement twitching in strains expressing heterologous *P. aeruginosa* pilins, suggesting TfpY and its homologues play a common role in pilus assembly. We showed that TfpY likely interacts with the major pilin and specific minor pilins but is not incorporated into the pilus itself. We propose that TfpY, along with the minor pilins at the pilus tip, primes pilus assembly. We identified two unique gain-of-function mutations in T4P regulatory genes that non-specifically restore twitching in *tfpY* mutants by increasing levels of cAMP and expression of T4P components. This study enhances our understanding of the complex functional and regulatory relationships between pilin and accessory proteins.

**IMPORTANCE:**

Type IV pili are surface filaments that enable versatile pathogens, like *Pseudomonas aeruginosa*, to adhere to and colonize surfaces. Pili are composed of diverse proteins called pilins, which serve as host receptors for phages. *P. aeruginosa* uses specific accessory proteins to glycosylate pilins to evade phage infection. Here, we show that TfpY is a conserved accessory protein that does not mediate phage defence. Instead, we propose a mechanism where TfpY facilitates efficient pilus assembly and function. A better understanding of TfpY function will provide insight into how its associated pilins have evolved to resist phage infection in the absence of post-translational modification, how some phages overcome this barrier to infection, and how this can guide the design of phage-based therapeutics.

## INTRODUCTION

Type IV pili (T4P) are widespread and functionally diverse prokaryotic surface filaments found in human, animal, and plant pathogens (1–3). *Pseudomonas aeruginosa*, a multidrug-resistant opportunistic pathogen, uses its T4P to facilitate motility, form drug-tolerant biofilms, and sense surfaces to upregulate the expression of other virulence factors (4–6). Due to their key role in host colonization, T4P serve as important therapeutic targets.

In *P. aeruginosa*, T4P are primarily composed of the major pilin subunit (PilA), a lollipop-shaped protein with a highly conserved N-terminal S-shaped α-helix and a more diverse C-terminal globular domain (7). PilA monomers in the inner membrane undergo rapid cycles of assembly and disassembly, forming a fiber that extends, attaches to a surface, and retracts (8, 9). The α1-N segment of the α1-helix anchors the pilins in the inner membrane, while the α1-C segment, the αβ-loop, a four-stranded antiparallel β-sheet, and the disulfide bonded-loop form the globular domain (10). Minor pilins and the adhesin protein PilY1 form the tip of the pilus, priming the incorporation of major pilins into the growing fiber (11, 12).

Aside from the conserved N-terminal α-helix which is buried inside the pilus fiber, major pilins share less than 25% pairwise amino acid sequence identity. Based on sequence and structural diversity, the major pilins of *P. aeruginosa* were previously categorized into five distinct groups (13). Except for group II strains, which include common lab strains mPAO1 and PAK, all other groups encode unique accessory proteins downstream of the pilin gene, forming a pilin-accessory gene cassette (13). A common feature of this cassette is a Rho-independent transcriptional terminator following the pilin gene, proposed to attenuate downstream accessory gene expression (7). A similar transcriptional terminator downstream of the major pilin gene has been described in other organisms that encode accessory proteins, including *Bacteroides nodosus*, *Eikenella corrodens*, and *Xanthomonas campestris* pv. vesicatoria, indicating it is likely a broadly conserved mechanism to control the level of accessory gene expression (14–16).

Group I and IV strains of *P. aeruginosa* encode unrelated glycosyltransferases, TfpO and TfpW, that add O-antigen units or homopolymers of α1,5-linked D-arabinofuranose, respectively, to distinct sites on their associated pilins (13, 17–20). Pilin glycosylation has been proposed to allow evasion of immune recognition and to protect *P. aeruginosa* from pilus-targeting bacteriophages (8, 21). The function of the remaining three accessory proteins TfpX, TfpY, and TfpZ, associated with groups IV, III, and V pilins, respectively, remains unclear (22). Co-expression of TfpX, TfpY, and TfpZ with their associated pilins *in trans* increased twitching motility when compared to *pilA* expression alone (22). However, mass spectrometry analyses showed that wild-type (WT) group III and V pilins are not post-translationally modified (22), and TfpX is not required for post-translational modification of group IV pilins in *P. aeruginosa* (23).

Here, we probe the role of TfpY and its homologues in T4P assembly and function in *P. aeruginosa*. Using the globally distributed strain *P. aeruginosa* PA14 as a model, we demonstrate that TfpY is a structurally conserved T4P accessory protein required for optimal pilus assembly and function. Unlike pilin glycosyltransferases TfpO and TfpW, TfpY does not provide phage defence. We show that TfpY expression is transcriptionally regulated with PilA and maintaining this regulation is important for cross-complementation of pilus function among TfpY-like proteins. Using chemical mutagenesis, we identified two gain-of-function mutations that increased twitching motility in *tfpY* mutants. These mutations, which mapped to the Pil-Chp regulatory system, likely restore motility by increasing cyclic AMP (cAMP) levels and thus overall expression of the T4P system. Finally, we showed that TfpY interacts with the major pilin and specific minor pilins but does not appear to be incorporated into the pilus. From these data, we propose that TfpY, along with the minor pilins at the tip of the pilus, facilitates recruitment of the first major pilin, allowing for subsequent addition of major pilins during pilus extension.

## RESULTS

### AlphaFold3 models suggest that TfpY-like proteins are structurally conserved among T4P-expressing bacteria

Previous bioinformatic analyses predicted that TfpY and related proteins (TfpX and TfpZ) have short cytoplasmic N-termini, three transmembrane helices, and periplasmic C-terminal globular domains (22). Sequence similarity among these proteins was concentrated in the transmembrane helices, while the diverse C-termini were predicted to provide pilin specificity, since the periplasmic C-termini of pilins are also variable. We used HHpred (24) to query representative protein sequences of TfpX, TfpY, and TfpZ to identify conserved domains and homologous proteins of known function. We noticed that TfpX in group IV strain Pa5196 was only 170 amino acids long, shorter than TfpY (235 amino acids) and TfpZ (245 amino acids). Strain PA7, another example of a group IV strain, encodes a TfpX homologue that is 244 amino acids long, more consistent with the lengths of TfpY and TfpZ. Therefore, both TfpX_Pa5196_ and TfpX_PA7_ were included in our analysis. Although HHpred can recognize glycosyltransferase domains in TfpO and TfpW, we were unable to confidently identify conserved domains or homologous proteins of known function for TfpY and its homologues.

**Fig 1:**
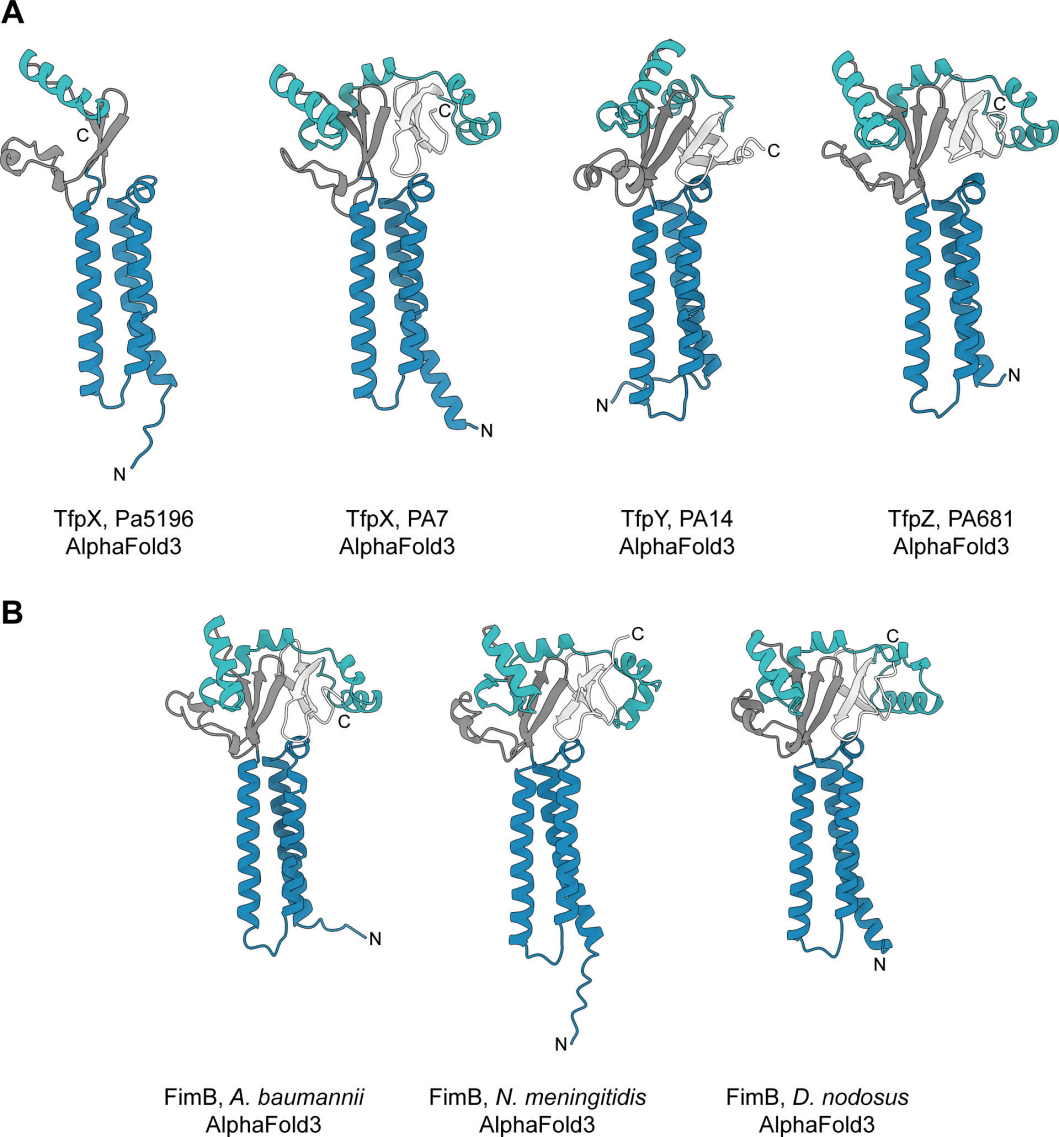
AlphaFold3 models suggest that TfpY-like proteins are structurally conserved among T4P-expressing bacteria. (**A**) Cartoon models of TfpY-like (FimB-like) proteins in *P. aeruginosa* (**B**) and other T4P-expressing bacteria (*A. baumannii*, *N. meningitidis*, and *D. nodosus*) in the phylum *Pseudomonadota*. With the exception of TfpX_5196_, all TfpY-like proteins have three N-terminal α-helices (blue), a three-stranded mixed β-sheet domain with a β2-α-β3 motif (dark grey), and an α-helix loop (teal) that extends into a three-stranded antiparallel β-sheet (light grey). All structures and error plots (Fig. S2A and B) were predicted using AlphaFold3 and modeled using UCSF ChimeraX.

We next aimed to determine if structural models of these proteins could inform protein function and used AlphaFold3 (25) to predict their structures. Except for the shorter TfpX_Pa5196_, all models shared three N-terminal α-helices, a three-stranded mixed β-sheet domain with a β2-α-β3 motif, and an α-helix that extends into a three-stranded antiparallel β-sheet (Fig. 1A). TfpX_Pa5196_ lacks the three-stranded antiparallel β-sheet found in TfpX_PA7_, even though both Pa5196 and PA7 encode TfpW homologues and group IV pilins. These structural differences may inform on which motifs are necessary and sufficient for TfpY-like protein function.

To explore evolutionary context and further understand the functional implications of these structural motifs, we used JackHmmer iterative search (26) to identify homologues of TfpX, TfpY, and TfpZ in the UniProtKB databases. Sequences sharing more than 50% sequence identity across the length of the proteins were clustered, and a representative sequence for each cluster was then aligned to generate a phylogenetic tree (Fig. S1). TfpY homologues, annotated as FimB in some species, were widely distributed in T4P-expressing bacteria in the phylum *Pseudomonadota*. AlphaFold3-predicted structural models of these homologues, including those from significant human, animal, and plant pathogens such as *Acinetobacter baumannii*, *Neisseria meningitidis*, and *Dichelobacter nodosus*, share architectural features with their *Pseudomonas* counterparts (Fig. 1B). While our comprehensive bioinformatic and structural analyses did not reveal potential function, they showcased the highly conserved architecture of TfpY-like proteins across diverse species, supporting the hypothesis that these proteins play a similar role.

### Conserving native transcriptional regulation of TfpY allows for cross-complementation of twitching

**Fig 2:**
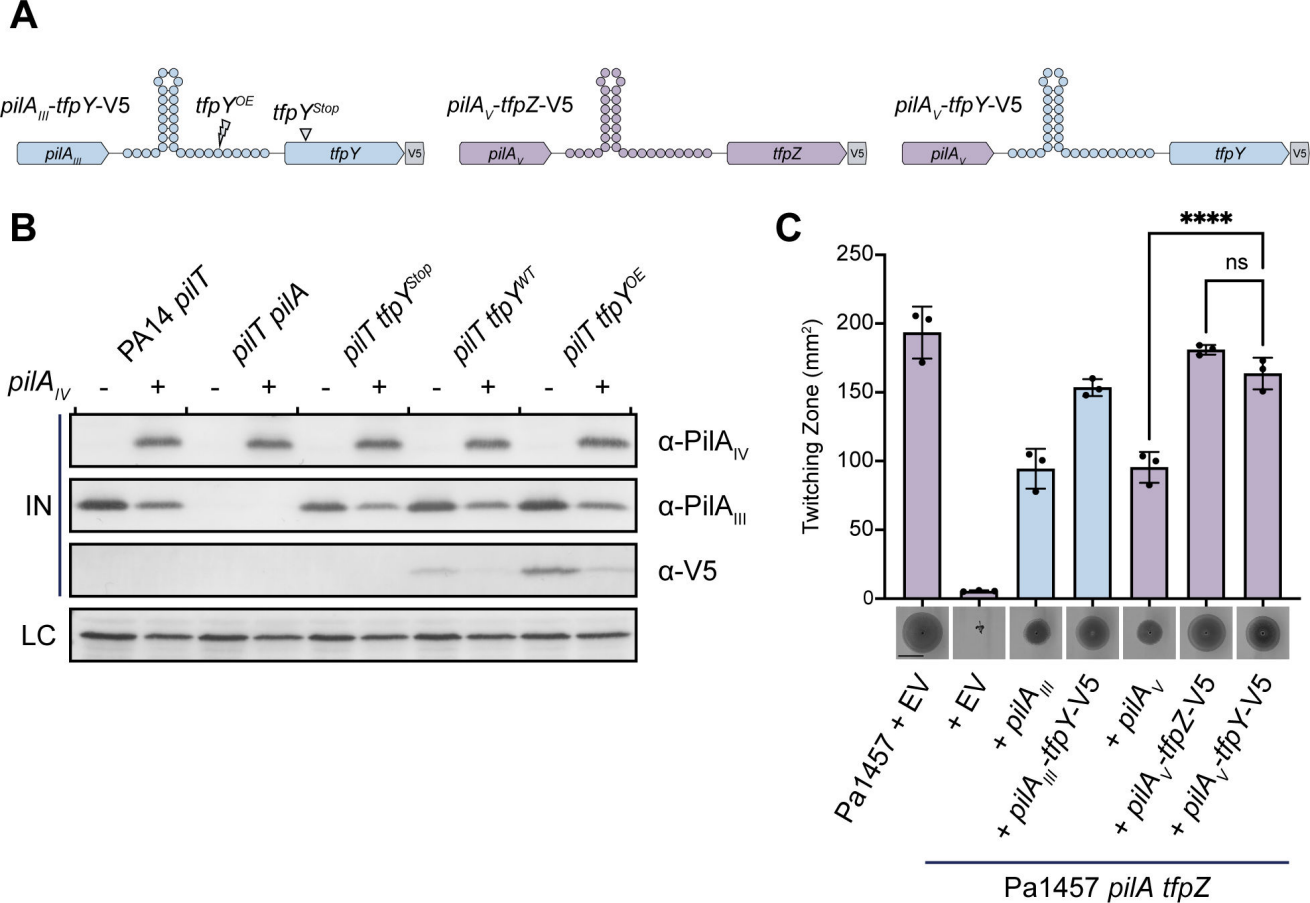
Conserving native transcriptional regulation of TfpY allows for cross-complementation of twitching. (A) Type IV pilin operon schematic for constructs used. Blue represents components from the PA14 group III pilin operon, while purple represents components from the Pa1457 group V pilin operon. (B) Overexpression of PilA_IV_ *in trans* reduces chromosomal PilA_III_ and TfpY-V5 levels. Western blots of whole-cell lysates were performed with α-V5, α-PilA_IV_, or α-PilA_III_ antisera. Flagellin levels were used as loading controls for α-V5, α-PilA_IV_, and α-PilA_III_ blots. All samples were induced using 0.1% L-arabinose. Blots are representative of three independent experiments. (C) TfpY can functionally complement group V pilins when integrated with its native transcriptional terminator sequence (*pilA_V_-tfpY*-V5). Purple bars represent recombinant expression of group V pilins, and blue bars represent recombinant expression of group III pilins. Twitching images are representative of three independent experiments. Scale bar represents 10 mm. ****P < 0.0001.

To test if TfpY-like proteins are functionally similar, Asikyan et al. (22) explored the cross-compatibility of TfpY (group III) and TfpZ (group V). They created a chimeric construct combining *pilA_V_* from *P. aeruginosa* strain 1457 and *tfpY* from PA14. When expressed *in trans* in a mPAO1 *pilA* mutant, the chimeric construct failed to increase twitching compared to *pilA_V_* alone, leading them to conclude that *pilA_V_* and *tfpY* were not functionally compatible and that accessory proteins are specific to their associated pilins. Unique transcription terminator elements found between pilin and associated accessory genes suggests they are co-expressed, and their stoichiometry is important (Fig. 2A). We hypothesized that failure to consider these regulatory elements when designing chimeric constructs likely altered the ratio at which *tfpY* was expressed compared to the native system, preventing complementation of twitching motility.

To inform our design of a new chimeric construct, we first tested if *pilA* and *tfpY* are co-expressed. PilA expression is regulated by a two-component system, PilSR (27, 28). PilS is a sensor kinase that monitors pilin levels in the inner membrane, and PilR is a response regulator whose phosphorylation by PilS in response to pilin depletion induces *pilA* expression (29). We expected that heterologous *pilA* expression would increase pilin levels in the inner membrane, thereby decreasing PilSR signalling and native pilin and accessory gene expression. Group IV *pilA* from strain Pa5196 was expressed in PA14 chromosomal deletions of *pilT*—encoding the retraction ATPase—that express no TfpY (*tfpY*^Stop^), WT levels of TfpY-V5 (*tfpY*^WT^), and greater than WT levels of TfpY-V5 (*tfpY*^OE^). TfpY-V5 expression levels were increased in the *tfpY*^OE^ mutant by introducing point mutations in the transcriptional terminator that were reported to increase read-through in other systems (30). A C-terminal V5 epitope tag was added to TfpY and its levels were visualized using Western blot. We showed that expression of PilA_IV_ *in trans* decreased both native PilA_III_ and TfpY levels (Fig. 2B). Interestingly, PilA_IV_ expression also decreased the levels of flagellin. Kilmury and Burrows (31) showed previously that PilR positively regulates expression of *fleSR*, encoding the FleSR two-component system that controls expression of genes required for flagellum biogenesis (32). Therefore, decreased PilA_III_ expression in response to ectopic PilA_IV_ likely reduces PilSR and FleSR activity. These data support a model where *pilA* and *tfpY* are co-expressed and the transcriptional terminator maintains an optimal stoichiometry for pilus assembly.

With new evidence that pilins and their accessory genes are co-expressed and that the transcriptional terminator is a key element of the cassette, we revisited cross-compatibility of TfpY-like proteins. We designed a chimeric construct that included the native transcriptional terminator from PA14 to maintain optimal ratios between the group V pilin gene *pilA_V_* and the group III accessory gene *tfpY* (Fig. 2A). We first showed that group V strain Pa1457 lacking *pilA* and *tfpZ* could assemble group III pilins and that *pilA_III_-tfpY* co-expression increased twitching, as demonstrated previously in group II strain mPAO1 (Fig. 2C). In contrast to previous findings, the new *pilA_V_-tfpY* chimeric construct restored twitching to a greater extent than *pilA_V_* alone, similar to expression of the native *pilA_V_-tfpZ* gene cassette (Fig. 2C). These data suggest that TfpY increases assembly of group V pilins when integrated with its native transcriptional terminator sequence. This result highlights the critical role of transcriptional regulation in the co-expression and functional compatibility of pilins and TfpY-like proteins in *P. aeruginosa*.

### TfpY expression promotes type IV pilus assembly and function

**Fig 3:**
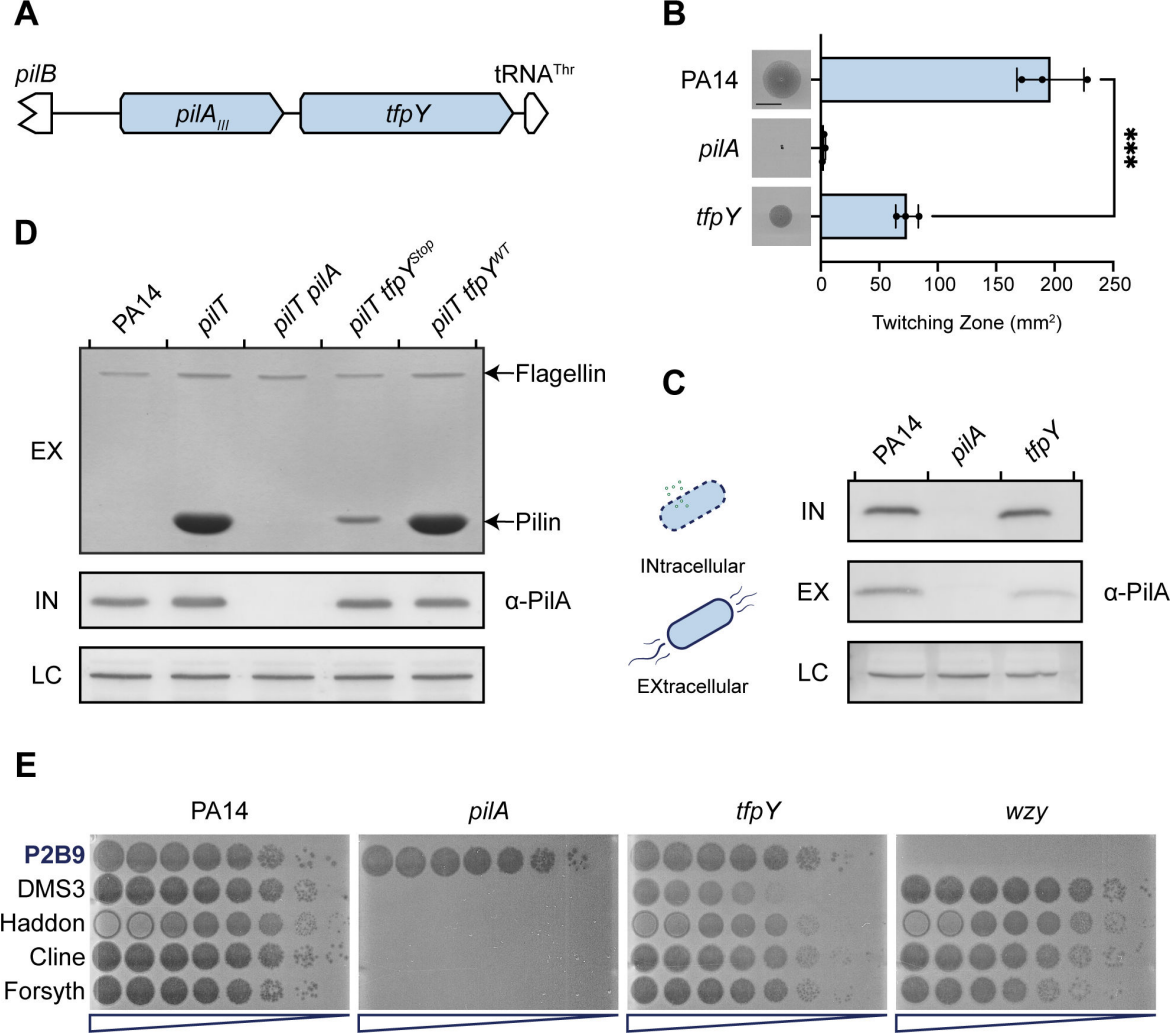
TfpY expression promotes type IV pilus assembly and function. (**A**) Type IV pilin operon in *P. aeruginosa* PA14. (**B**) *tfpY* mutants twitch less than WT. Twitching images are representative of three independent experiments. Scale bar represents 10 mm. ***P < 0.0005. (**C**) Intracellular pilin levels are similar between WT and *tfpY* mutants; however, *tfpY* mutants display decreased surface piliation. Western blots of whole-cell lysates, which represent intracellular protein levels (IN), and sheared surface proteins samples, which represent extracellular protein levels (EX), were analyzed using α-PilA_III_ antisera. Flagellin levels were used as loading controls (LC) for α-PilA_III_ blots. Blots are representative of three independent experiments. (**D**) TfpY expression increases surface piliation in *pilT* mutants. Sheared surface protein samples, which represent extracellular protein levels (EX), were analyzed using a Coomassie-stained SDS-PAGE gel. Western blots of whole-cell lysates, which represent intracellular protein levels (IN), were analyzed using α-PilA_III_ antisera. Flagellin levels were used as LC for α-PilA_III_ blots. Gels and blots are representative of three independent experiments. (**E**) TfpY expression increases phage susceptibility. Serially diluted stocks of phage P2B9 (LPS-specific control, bolded) and T4P-specific phages DMS3, Haddon, Cline, and Forsyth were spotted onto top agar inoculated with the indicated strain. Phage plaquing assay is representative of three independent experiments.

We next took experimental approaches to address the question of TfpY function. We focused on TfpY in the well-studied *P. aeruginosa* strain PA14, as a representative member of the TfpY-like family of proteins (Fig. 3A) (13, 33). Consistent with previous reports, *tfpY* mutants had decreased twitching motility compared to WT, which could be due to changes in pilus assembly or intracellular pilin levels (Fig. 3B). Unlike other common lab strains such as mPAO1, we recovered very low levels of surface pili from PA14 in sheared surface protein assays (Fig. S3A). However, we recovered substantial amounts of surface pili from PA14 retraction-deficient mutants and observed comparable levels of twitching between mPAO1 and PA14, showing that PA14 pili are fully functional despite being difficult to shear (Fig. S3A and B). Using multilocus sequence typing (34), we identified two additional PA14-like isolates in the Wright Clinical Collection (WCC) and quantified their surface piliation and twitching levels. WCC strains C0072 and C0366 displayed increased twitching compared to mPAO1, but again, we recovered little to no surface pili from these strains, similar to PA14 (Fig. S3A and B). Therefore, this surface piliation phenotype is generalizable to other PA14 isolates. To overcome this inability to visualize sheared surface pili using Coomassie-stained gels, Western blot analysis was used to assess both sheared surface proteins and whole-cell lysates. We showed that levels of intracellular pilins are similar in WT and a *tfpY* mutant, but *tfpY* mutants have decreased surface piliation (Fig. 3C), consistent with their decreased motility.

Given that TfpY expression promotes twitching and surface piliation, we investigated whether TfpY-mediated increases in surface piliation resulted from changes in pilus assembly. We previously tested this hypothesis using recombinant expression of TfpY in a PA14 retraction-deficient background (*pilT::*FRT) (22). We showed that stoichiometry of TfpY to PilA is important and that copy number effects from plasmid-based expression can bias phenotypes (35, 36). Here, using chromosomal deletions of *pilT*, we showed that in the absence of pilus retraction, TfpY expression substantially increased the level of recoverable surface pili, indicating that reduced twitching observed in *tfpY* mutants resulted from decreased pilus assembly (Fig. 3D). These results confirmed our previous plasmid-based expression data and demonstrated that the V5 tag had no impact on TfpY function.

Pilin accessory proteins TfpO (group I) and TfpW (group IV) glycosylate their associated pilins to evade pilus-specific phage binding (21). To determine if TfpY plays a role in pilus-specific phage defence, we tested the susceptibility of PA14 WT versus a *tfpY* mutant to four pilus-specific phages—DMS3, Haddon, Cline, and Forsyth—as well as a control lipopolysaccharide (LPS)-specific phage, P2B9. Phages were serially diluted and spotted onto bacterial lawns of PA14 WT or a *tfpY* mutant, as well as PA14 mutants lacking *pilA* or *wzy*, an LPS biosynthesis gene, as controls for receptor specificity. Depending on the phage, we observed a 10- to 1,000-fold decrease in susceptibility of the *tfpY* mutant strain (Fig. 3E), with DMS3 infectivity showing the largest decrease of three logs. Therefore, in contrast to TfpO and TfpW, TfpY expression increases phage susceptibility, likely because it increases pilus assembly and thus receptor availability.

### Modulating surface piliation causes phage-specific differences in susceptibility

**Fig 4:**
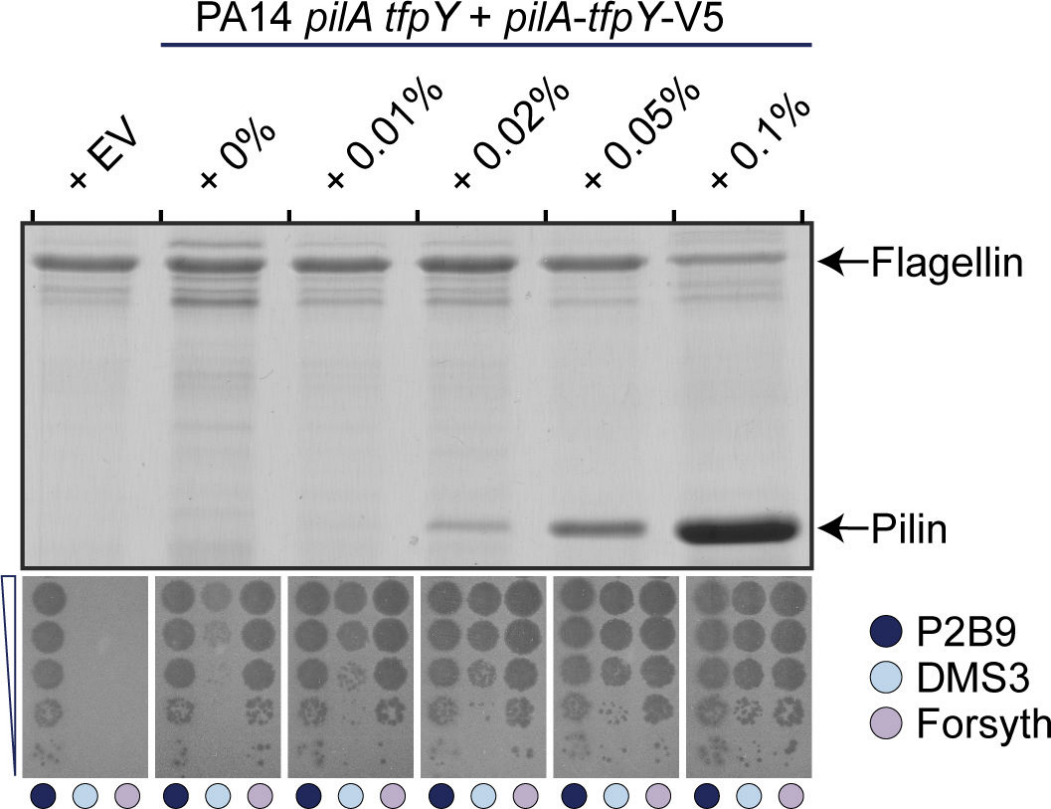
Modulating surface piliation causes phage-specific differences in susceptibility. Increasing the levels of L-arabinose increased surface piliation. Flagellin levels were used as loading controls. Coomassie-stained SDS-PAGE gel is representative of three independent experiments. Serially diluted stocks of phages P2B9, DMS3, and Forsyth were spotted onto top agar inoculated with the indicated strain and increasing levels of L-arabinose. Phage plaques for dilutions 10^−3^ to 10^−7^ are shown. Phage plaquing assay is representative of three independent experiments.

As T4P are among the most common receptors for tailed *P. aeruginosa* phages, we wanted to understand if DMS3 infectivity was more sensitive to changes in surface piliation compared to other phages. We complemented a PA14 *pilA tfpY* double mutant with *pilA-tfpY*-V5, which includes the transcriptional terminator to ensure the correct stoichiometry, in an arabinose-inducible vector. *pilA-tfpY*-V5 expression was induced using arabinose concentrations from 0% to 0.1%. In a sheared surface protein assay, increasing the levels of arabinose increased surface piliation (Fig. 4).

Next, we tested the susceptibility of these recombinant strains to phages DMS3 and Forsyth, as well as the LPS control phage, P2B9 (Fig. 4). While some pilus-specific phages like Forsyth showed infectivity levels similar to our LPS control phage, even at 0% arabinose—likely due to leaky expression from the arabinose-inducible promoter (37)—other phages, such as DMS3, showed a direct correlation between surface piliation and phage susceptibility. Therefore, there are phage-specific differences in susceptibility in response to changes in the levels of surface piliation.

### TfpY may interact with major and minor pilins but cannot be detected in assembled pili

**Fig 5:**
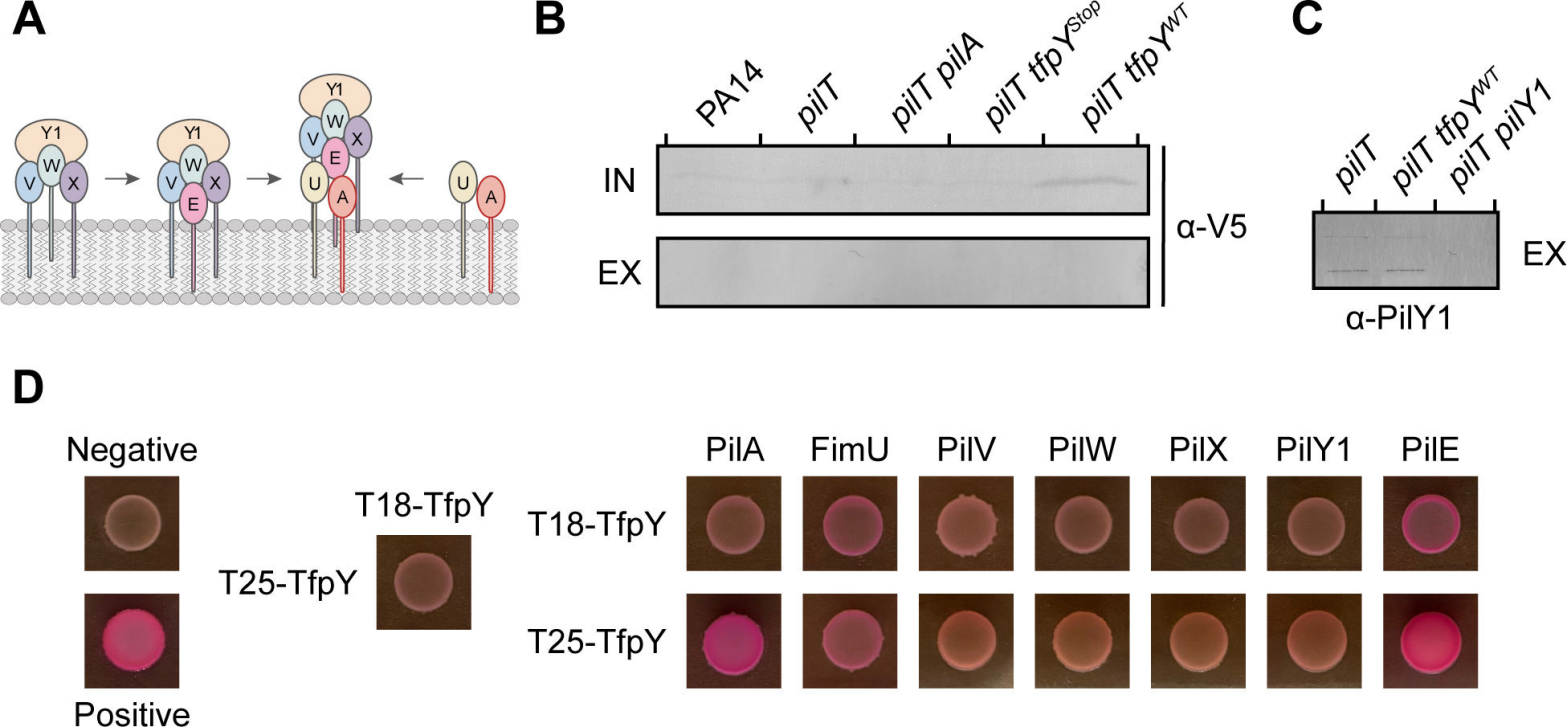
TfpY may interact with major and minor pilins but cannot be detected in assembled pili. (**A**) Minor pilins PilVWX and PilY1 form an initiation complex in the inner membrane, priming subsequent addition of PilA via the adaptor minor pilins PilE and FimU (11). (**B**) Western blot analysis of *pilT* mutants showed that TfpY cannot be detected in extracellular protein fractions. Western blots of whole-cell lysates, which represent intracellular protein levels (IN), and sheared surface proteins samples, which represent extracellular protein levels (EX), were analyzed using α-V5. Blots are representative of three independent experiments. (**C**) PilY1 can be detected in extracellular protein fractions. Western blots of sheared surface proteins samples, which represent extracellular protein levels (EX), were analyzed using α-PilY1 antisera. Blots are representative of three independent experiments. (**D**) Positive signal between PilA and the minor adaptor pilins FimU and PilE with T18-TfpY and/or T25-TfpY fusions was observed using BACTH. Empty pUT18C and pKNT25 plasmids were used as negative controls, while pUT18C-PilS and pKNT25-PilS were used as positive controls. Plates were incubated for 48 h at 30°C. All samples were induced using 0.5-mM IPTG. Images are representative of three independent experiments.

Like TfpY, the minor pilins and the non-pilin adhesin PilY1 are required for pilus assembly. Our current model of assembly proposes that minor pilins PilVWX and PilY1 form an initiation complex in the inner membrane, priming subsequent addition of PilA via the adaptor minor pilins PilE and FimU (Fig. 5A) (11). Given its association with PilA, we tested whether TfpY, like the minor pilins, may help to prime pilus assembly and possibly be incorporated into the pilus fiber. Using retraction-deficient backgrounds, we tested strains expressing no TfpY or WT levels of TfpY-V5 for its presence in assembled pili. While TfpY-V5 was not detected in the extracellular protein fractions via Western blot (Fig. 5B), we showed that we could detect PilY1, which is incorporated as a single copy per pilus, using the same amount of protein sample (Fig. 5C). Consistent with previous reports, both full-length and a truncated fragment of PilY1 were detected in the sheared surface protein sample in PA14 *pilT* but were absent in PA14 *pilT pilY1* (11).

We hypothesized that TfpY may instead contribute to pilus assembly by promoting interactions of PilA with the minor pilin complex in the inner membrane. To test if TfpY interacts with itself, PilA, and components of the minor pilin complex, we used a bacterial adenylate cyclase two-hybrid assay (BACTH) (38). Fusions of TfpY, PilA, minor pilins, and PilY1 to the T18 fragment were co-expressed with T25-TfpY in *Escherichia coli* BTH101 (as well as the reverse, with TfpY fused to T18 and potential partners to T25), with interactions detected on MacConkey-maltose agar plates. We observed a positive signal when T25-TfpY was co-expressed with T18-PilA, while the inverse combination, T18-TfpY and T25-PilA, did not produce a detectable signal (Fig. 5D). Co-expression of TfpY and PilE produced a positive signal, although this interaction was weaker when T18-TfpY was paired with T25-PilE compared to T25-TfpY paired with T18-PilE. A weak interaction was also observed between TfpY and FimU across both fusion configurations.

To address the limitations of expressing fusion proteins in a heterologous system, additional negative controls were included, such as the non-T4P-associated inner membrane proteins, PelD and PelE, which are involved in Pel polysaccharide biogenesis (39). As expected, co-expression of PelD and PelE produced a strong positive signal (Fig. S4) (39). A weak positive signal was also detected between T18-TfpY and T25-PelD but not the reverse, T25-TfpY and T18-PelD. There was no signal for TfpY and PelE in either configuration. Due to the weak asymmetric signal between TfpY and PelD, weak positive interactions observed in our BACTH assays should be interpreted with caution. Collectively, these findings suggest that while TfpY may not be incorporated into the pilus fiber, it could interact with major and adaptor minor pilins to promote assembly at the initiation step.

### Gain-of-function mutations restore twitching motility in *tfpY* mutants

**Fig 6:**
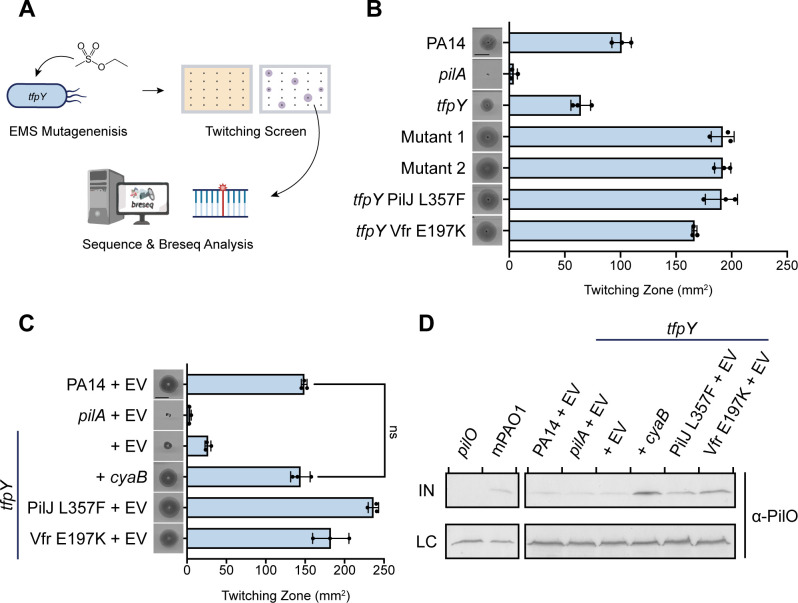
Gain-of-function mutations restore twitching motility in *tfpY* mutants. (**A**) Schematic of EMS mutagenesis protocol. (**B**) Using EMS at 100 mM, two gain-of-function mutants were generated that displayed increased twitching compared to a PA14 *tfpY* mutant. Two unique mutations of interest were identified following breseq analysis—PilJ L357F and Vfr E197K (Mutants 1 and 2). These mutations were regenerated in a clean PA14 *tfpY* background to confirm the phenotypes. (**C**) CyaB expression *in trans* in a *tfpY* mutant restored twitching motility to WT levels. Twitching images are representative of three independent experiments. Scale bar represents 10 mm. (**D**) Elevated PilO levels were observed in a *tfpY* mutant complemented with *cyaB* and in the gain-of-function mutants PilJ L357F and Vfr E197K, compared to WT, *pilA*, and the *tfpY* mutant. Western blots of whole-cell lysates, which represent intracellular protein levels (IN), were analyzed using α-PilO_II_ antisera. Non-specific bands were used as loading controls (LC). Blots are representative of three independent experiments.

Although most *P. aeruginosa* strains encode pilins with accessory proteins, group II strains such as mPAO1 and PAK can efficiently assemble functional pili in the absence of accessory proteins (13). To gain further insight into why group III and related strains require accessory proteins, we conducted a gain-of-function screen in a *tfpY* mutant background. Ethyl methanesulfonate (EMS) chemical mutagenesis was used to increase the frequency of isolating mutants with increased twitching motility in the absence of *tfpY* (Fig. 6A) (40).

From this screen, we identified two mutants with increased twitching compared to a PA14 *tfpY* mutant and unexpectedly, WT PA14 (Fig. 6B). Whole genome sequencing and comparison of the mutants to the parent strain using breseq analysis (41) led to the identification of two functionally related mutations of interest, PilJ L357F and Vfr E197K, which were regenerated in a clean PA14 *tfpY* mutant background to confirm their phenotypes (Fig. 6B). Both mutations are associated with the Pil-Chp system that modulates levels of the secondary messenger cAMP (42). PilJ encodes the membrane-bound methyl-accepting chemotaxis protein that transduces a poorly defined signal to activate the CyaB adenylate cyclase (42), while Vfr is a cAMP-binding regulator homologous to *E. coli* CRP (43). Since expression of multiple T4P genes is positively regulated by Vfr in response to high levels of cAMP (44), this result suggested that the increase in twitching was due to an overall increase in T4P components, rather than a TfpY-specific mechanism.

To test if an increase in cAMP increases twitching motility independently of TfpY, we expressed CyaB *in trans* from a multicopy plasmid in a *tfpY* mutant, which restored twitching to WT levels (Fig. 6C). Using Western blots, we measured PilO levels in our *cyaB*-complemented *tfpY* and gain-of-function mutants. PilO, a highly conserved core component of the T4P assembly machinery, serves as an indirect indicator of the overall abundance of T4P components. PilO levels were increased in both the *tfpY* mutant complemented with *cyaB* and in the *tfpY* gain-of-function mutants PilJ L357F and Vfr E197K, compared to WT, *pilA*, and the *tfpY* mutant (Fig. 6D). These data suggest that group III strains like PA14 depend on accessory proteins such as TfpY for efficient pilus assembly, but elevated cAMP levels can bypass this requirement by globally increasing the expression of T4P components to restore twitching motility.

## DISCUSSION

*P. aeruginosa* is a well-studied model organism for T4P research and a versatile opportunistic pathogen (45). Based on sequence and structural diversity, the major pilins of *P. aeruginosa* were categorized into five distinct groups (groups I–V) (13). Here, we characterized the functional role of group III pilin accessory protein TfpY in *P. aeruginosa* PA14. We showed that TfpY, unlike pilin glycosyltransferases TfpO and TfpW, does not contribute to pilus-specific phage defence. Instead, TfpY’s primary role appears to be facilitating T4P assembly. We showed that TfpY may interact with PilA and minor pilins FimU and PilE but is not incorporated into the pilus fiber itself. Its inability to be incorporated into the pilus fiber is consistent with the predicted structure of TfpY and its homologues, with three transmembrane segments instead of the single segment present in pilin proteins. We propose that TfpY functions as a chaperone or adapter to enhance interactions of PilA with the minor pilins, aiding in the initiation of pilus assembly.

The presence of specific accessory genes is among the characteristics used to differentiate *pilA* variants in *P. aeruginosa* (13). This approach is effective for group I and IV strains, where accessory genes *tfpO* and *tfpW* encode structurally distinct proteins that use different glycan substrates for post-translational modification of pilins (13, 17–20). However, our bioinformatic analyses showed that, with the exception of truncated TfpX_Pa5196_, AlphaFold3 models of TfpX_PA7_, TfpY, and TfpZ share a highly conserved architecture. The absence of the three-stranded antiparallel β-sheet in the TfpX homologue from Pa5196, compared to PA7, suggests that this motif may be critical for the function of TfpY-like proteins in some group IV strains but not others. Given that most TfpW-encoding species lack TfpX homologues and TfpX is not required for pilin glycosylation in Pa5196 (23), TfpX_Pa5196_ may have become dispensable in this strain. Due to the predicted conserved architecture of TfpY-like proteins, other characteristics such as pilin sequence, overall length, and the length of the C-terminal disulfide-bonded loop are more reliable indicators of PilA type in *P. aeruginosa*.

Models of TfpY-like proteins from diverse species, including significant human, animal, and plant pathogens, share conserved motifs, supporting our hypothesis that these proteins perform similar functions. To test this hypothesis, we used a chimeric construct combining a group V pilin gene with the group III accessory gene *tfpY* and its transcriptional terminator to restore twitching motility to levels similar to the native group V pilin-*tfpZ* cassette. These findings challenge our previous assumption of specificity in pilin-accessory protein interactions (22), suggesting functional cross-complementation can occur if key regulatory elements such as transcriptional terminators are properly maintained. Future work should explore the extent of pilin variability that can be tolerated while maintaining accessory protein and T4P machinery compatibility, as these factors could impact the successful horizontal acquisition of new pilin-accessory gene cassettes. This question was addressed in part by Watson et al. (46), who showed that the major pilin FimA from *D. nodous* expressed in group II *P. aeruginosa* PAK and PAO1 *pilA* mutants could support limited twitching and phage infectivity. It would be interesting to test whether co-expression of FimA and TfpY can provide functional cross-complementation, or if the TfpY-like protein FimB from *D. nodous* can cross-complement twitching when co-expressed with a group III *Pseudomonas* pilin. Such studies could provide further insight into the mechanism by which TfpY-like proteins facilitate T4P assembly, identify critical structural elements required for cross-compatibility, and address questions about co-evolution of pilins and accessory proteins across diverse bacterial species.

Our experimental data support previous findings that TfpY is important for the assembly of functional T4P in *P. aeruginosa*. TfpY’s role is different from other pilin accessory proteins TfpO and TfpW, which are involved in pilin glycosylation and phage defence (21). Instead, TfpY modulates susceptibility to certain pilus-specific phages by optimizing pilus assembly, highlighting a novel aspect of pilin accessory protein function. Further studies that address how infectivity of some phages is correlated with piliation levels could provide valuable insights into the complex interplay between pilus dynamics and phage susceptibility. This concept is particularly relevant given growing interest in phage therapy as a strategy to combat antibiotic-resistant infections (47, 48).

The increase in phage susceptibility linked to TfpY expression raises the question of why TfpY is conserved in T4P-expressing bacteria, since many bacteria can assemble functional pili without pilin accessory proteins. We identified twitching gain-of-function mutations in *tfpY* mutants that mapped to PilJ (L357F) and Vfr (E197K). PilJ along with other members of the Pil-Chp system upregulate cAMP synthesis upon sensing a surface (49). Vfr, a transcription factor, binds cAMP to positively regulate virulence factor expression, including transcription of the two-component system, FimS-AlgR, which regulates expression of the minor pilin operon (50). Based on structural modeling, we predict that PilJ L357F is located in the cytoplasmic domain of PilJ, while Vfr E197K is in the C-terminal DNA-binding domain of Vfr (Fig. S5) (51, 52). Our results suggest that these mutations likely increase cAMP levels, increasing T4P machinery biogenesis and twitching motility. We showed that CyaB overexpression in a *tfpY* mutant restores twitching to WT levels and that PilO levels, an indirect indicator of the overall abundance of T4P components, were elevated in gain-of-function mutants. Together, these data suggest that elevated cAMP can compensate for loss of TfpY by increasing overall expression of T4P machinery.

The specific structural and functional characteristics of group III pilins may offer additional context for understanding the requirement for accessory proteins. Group III and V strains encode a different set of minor pilins than groups I, II, and IV (53), and their pilins are larger and structurally more complex than those of groups I and II (54, 55). Group III pilins may have evolved, or been horizontally acquired, to evade infection from some pilus-specific phages. Consequently, an adaptor protein may be required to mediate interactions between such pilins and the highly conserved T4P assembly system. The low abundance of TfpY and its potential interaction with major and specific minor pilins FimU and PilE suggest that TfpY acts at the crucial first step of assembly, facilitating recruitment of a major pilin to the priming complex. Further increasing TfpY levels by mutating the transcriptional terminator did not increase surface piliation, which suggests that TfpY alone is unable to increase pilus assembly. Instead, initiation of assembly is likely restricted by limited availability of minor pilin complexes and T4P machines. Finally, the absence of TfpY in the extracellular pilus fraction despite its potential interactions with pilins in the inner membrane supports our model that while TfpY functions in the early stages of pilus assembly, it is not a structural component of the pilus.

This study advances our understanding of the role of TfpY-like proteins in T4P assembly and function. Due to the broad distribution of TfpY-like proteins in important pathogens, insights into TfpY-optimized pilus assembly can inform the design of pilus-targeting vaccines, phage cocktails, or small-molecule inhibitors that target multidrug-resistant bacteria.

## MATERIALS AND METHODS

### Phylogenetic analyses

TfpX_5196_ (AAM52061), TfpX_PA7_ (WP_124179916), TfpY_PA14_ (ABJ13791), and TfpZ (WP_023110409) protein sequences were used to iteratively search the UniProtKb database using Jackhmmer (26). Protein sequences were downloaded and clustered using mmseqs (version15.6f452) using the “easy-cluster” option and the “–min-seq-id 0.5 cov-mode 0” parameters (56). Sequences shorter than 170 amino acids were removed, followed by multiple-sequence alignment by Clustal Omega (version 1.2.3) using default parameters (57). IQ-TREE (version 2.2.6) (58) was used to generate a phylogenetic tree from the multiple-sequence alignment using the “-m LG -nt AUTO -nm 5000 -bb 1000” parameters, followed by phylogenetic tree visualization using iTOL (version 6) (59).

### Protein modeling

AlphaFold3 was used to predict protein structures and generate error plots. AlphaFold3-predicted structures, the X-ray crystal structure of Vfr (PDB accession number 2OZ6), and error plots were visualized using UCSF ChimeraX (60).

### Bacterial strains and growth conditions

Bacterial strains were grown overnight at 37°C in lysogeny broth (LB) (BioShop) at 200 rpm or on 1.5% LB agar plates from glycerol stocks stored at −80°C. Liquid and solid media were supplemented with antibiotics at the following final concentrations (μg/mL) when necessary: 100-µg/mL carbenicillin (Carb), 15-µg/mL gentamicin (Gm), 50-µg/mL kanamycin (Kan) for *E. coli*, and 30-µg/mL Gm for *P. aeruginosa*. L-arabinose at concentrations of 0.01% to 0.2% was used to induce expression of the pBADGr and pHERD30T ara promoter in recombinant strains. The complete list of bacterial strains and plasmids used in this study is provided as Table S1.

### Molecular biology

All primers used in this study are listed in Table S2. A DNA fragment containing *pilA*, the stem-loop transcriptional terminator region, and V5-tagged *tfpY* from PA14 was synthesized (gBlock, IDT). Site-directed mutagenesis using overlap extension polymerase chain reaction (PCR) was used to introduce stabilizing cytosine point mutations within the transcriptional terminator region of *pilA* and *tfpY* to create *pilA-tfpY*^OE^ (cytosines at T2 and T3 of U-stretch) (30). A premature stop codon at Leu59 in *tfpY* was introduced using site-directed mutagenesis by overlap extension PCR to generate *pilA-tfpY*^Stop^. Additional genes of interest were amplified using primers flanking the gene of interest including the native ribosome binding site from genomic or plasmid DNA. DNA fragments were cloned into arabinose-inducible vectors pBADGr and pHERD30T using standard restriction digest and ligation methods. Plasmids were introduced into chemically competent *E. coli* by heat shock or by electroporation into *P. aeruginosa*.

Primers listed in Table S2 were used to amplify mature PilA, minor pilins, and PilY1 from PA14 chromosomal DNA. All constructs were cloned into pUT18C and pKNT25 using standard restriction digest and ligation methods.

PA14 *tfpY* chromosomal mutant constructs were created by combining existing mutant constructs with a synthetic DNA fragment that included V5-tagged *tfpY* and 500-bp downstream of the *tfpY* gene using overlap extension PCR (gBlock, IDT). To generate the EMS mutant knock-in constructs, primers flanking ~1-kb upstream and downstream each mutation of interest were used to amplify the mutation-containing region from chromosomal DNA. Chromosomal deletion constructs were generated using methods previously described (61). All constructs were cloned into suicide vector pEX18Gm using standard restriction digest and ligation methods. Chromosomal mutants were generated using allelic exchange, as previously described (61). Plasmids were confirmed by Sanger sequencing (McMaster Genomics Facility) or by Oxford Nanopore Technology whole-plasmid sequencing (Plasmidsauras).

### Twitching motility assays

Twitching motility was tested as previously described (62). In brief, overnight single colonies were stab-inoculated in triplicate to the bottom of cell culture-treated Nunc OmniTray Single-Well Plates (ThermoFisher Scientific) containing 1% LB agar, supplemented with L-arabinose and Gm when necessary. After inoculation, plates were incubated at 37°C for 16–18 h. Agar was carefully removed after incubation and twitching zones were stained with 1% crystal violet for 10 min. Plates were rinsed with distilled water to remove excess dye and air-dried. Plates were imaged using a flatbed scanner and twitching zone area was measured using ImageJ (63).

### Sheared surface protein analysis

Sheared surface proteins were collected as previously described with modifications (22). Briefly, strains were streaked in a grid-like pattern on 1.5% LB agar plates containing L-arabinose and Gm when necessary. Two plates per sample were incubated overnight at 37°C. Bacteria from both plates were gently scraped from the agar surface using an inoculation loop and resuspended in 3 mL of 1X sterile phosphate-buffered saline (PBS, pH 7.4). 1× PBS was diluted from a 10× PBS stock (80-g NaCl, 2-g KCl, 26.8-g Na_2_HPO_4_-7H_2_O, and 2.4-g KH_2_PO_4_ in 1-L Milli-Q H_2_O, pH 7.4). Cells were vortexed vigorously for 30 s to shear surface-exposed pili and flagella. The cell suspension was separated into two 1.5-mL microcentrifuge tubes and centrifuged at 21,000 × *g* for 1 h. The supernatant was transferred into two new 1.5-mL microcentrifuge tubes, followed by the addition of 1-M MgCl_2_ at a final concentration of 100 mM. Microcentrifuge tubes were vortexed briefly, followed by protein precipitation overnight at 4°C. Precipitated proteins were harvested by a 30-min centrifugation at 21,000 × *g*. The supernatant was decanted and the pellet resuspended in 25–50 μL of 1× sodium dodecyl sulfate-polyacrylamide gel electrophoresis (SDS-PAGE) sample loading dye (250-mM Tris, pH 6.8, 5% β-mercaptoethanol, 40% glycerol, 8% SDS, and 0.02% bromophenol blue). The samples from both microcentrifuge tubes were pooled and boiled for 10 min, centrifuged for 10 min at 21,000 × *g*, and then resolved on 15% SDS-PAGE gels at 120 V in 1X Tris-glycine running buffer with a prestained protein ladder (BLUelf). 1× Tris-glycine running buffer was diluted from a 10× stock (30.3g Tris, 144-g glycine, and 20-mL 10% SDS in 1-L Milli-Q H_2_O). Coomassie blue dye was used to visualize protein bands.

### Western blot analysis

After vortexing cells to shear surface pili and flagella, the remaining cells were resuspended in sterile 1× PBS to a final optical density (OD_600_) of 0.6. One milliliter of this standardized culture was transferred to a microcentrifuge tube, and cells were pelleted at 21,000 × *g* for 10 min. The supernatant was removed, and the pelleted cells were resuspended in 50 µL of 1× SDS-PAGE sample loading dye. Samples were boiled for 10 min, centrifuged for 10 min at 21,000 × *g*, and then resolved on 15% SDS-PAGE gels at 120 V in 1× Tris-glycine running buffer with a prestained protein ladder (BLUelf). After SDS-PAGE, proteins were transferred to a 0.45-µm nitrocellulose membrane (BioRad) for 1 h at 225 mA in 1× transfer buffer (20% methanol and 100-mL Tris-glycine buffer stock without SDS in 1-L Milli-Q H_2_O). Membranes were blocked with 5% skim milk (BioShop) resuspended in 1× Tris-buffered saline (TBS, pH 7.6) for 2 h at room temperature. 1× TBS was diluted from a 10× stock (24-g Tris and 88-g NaCl in 1-L Milli-Q H_2_O, pH 7.6). For detection of TfpY, blots were incubated with 1/1,000 dilution (1× TBS) of α-V5 polyclonal primary antibodies produced in rabbit (Sigma-Aldrich). For detection of PilA_III_, PilA_IV_, PilY1, and PilO_II_ blots were incubated with 1/1,000 dilution (1× TBS) of rabbit polyclonal antibodies, as described previously (13, 64, 65). Blots were incubated with primary antibodies overnight at room temperature. Membranes were washed with 1× TBS and then incubated with 1/3,000 dilution (1× TBS) of goat α-rabbit alkaline phosphatase-conjugated secondary antibodies for 1 h at room temperature (BioRad). Membranes were washed with 1× TBS and then developed using 5-bromo-4-chloro-3-indolylphosphatase and nitro blue tetrazolium chloride (BioShop) resuspended in alkaline phosphatase buffer (1-mM Tris, 100-mM NaCl, and 5-mM MgCl_2_, pH 9.5).

### Phage isolation and genome sequence analysis

Stocks of *Pseudomonas* pilus-specific phages Haddon and Cline were obtained originally from the Félix d'Hérelle Reference Center for Bacterial Viruses at Université Laval labeled as “Lindberg F10” and “Lindberg 68,” respectively, while we isolated phage Forsyth from wastewater. All were amplified using *P. aeruginosa* PA14 as the host strain and confirmed to be pilus-specific using a *pilA* mutant. Phages were plaque purified three times, and re-amplified on PA14, followed by genomic DNA extraction (Norgen Biotek Phage DNA Isolation Kit) and Illumina sequencing at SeqCenter (Pittsburgh, PA, USA). The resulting reads were assembled into contigs using SPAdes (3.15.5) (66) and annotated using Pharokka (1.7.3) (67). Annotated sequences were submitted to GenBank and are available at the following accession numbers: PQ571159 (*Pseudomonas* phage Forsyth), PQ571160 (*Pseudomonas* phage Cline), and PQ571161 (*Pseudomonas* phage Haddon). Based on their sequences, Forsyth, Cline, and Haddon are novel phages. Forsyth is ~84% similar to JBD69 (NC_030908), and Cline is ~85% similar to JBD-93 (NC_030918) and MP22 (NC_009818), while Haddon has essentially no similarity to sequenced phages.

### Phage plaquing assays

Overnight cultures of bacteria were subcultured 1:100 in LB media containing Gm when necessary and grown for 3–5 h at 37°C with shaking (200 rpm). Subcultures were standardized to a final OD_600_ of 0.3 in LB media, and 120 µL of this diluted culture was mixed with 12 mL of 0.6% LB agar and poured onto cell culture-treated Nunc OmniTray Single-Well Plates or standard petri dishes. LB agar was air-dried with the lid off in a biosafety cabinet for 40 min. Phage stocks were standardized to 10^10^ pfu/mL and serially diluted (10^−1^ to 10^−8^) in phage buffer (10-mM CaCl_2_, 10-mM MgSO_4_, 68-mM NaCl, and 10-mM Tris-HCl, pH 7.5). Five microliters of each dilution was spotted onto the prepared plates. Phage spots were air-dried with the lid on. Plates were incubated inverted for 18 h at 37°C or at room temperature for 48 h. Plates were imaged using a flatbed scanner and phage titer was estimated using the lowest dilution with visible individual plaques.

### Bacterial two-hybrid analysis

*E. coli* BTH101 was co-transformed with pUT18C and pKNT25 plasmids expressing a protein of interest fused to T18 and T25 domains of adenylate cyclase, respectively. Empty pUT18C and pKNT25 plasmids were used as negative controls, while pUT18C-PilS and pKNT25-PilS were used as positive controls (29). *E. coli* BTH101 was grown overnight at 37°C in LB supplemented with Carb and Kan at 200 rpm. Overnight cultures were diluted to an OD_600_ of 0.6 using LB media. Four microliters of standardized cultures were spotted onto MacConkey agar indicator media supplemented with Carb, Kan, 1% maltose, and 0.5-mM isopropyl β-D-1-thiogalactopyranoside (IPTG) that had been air-dried with the lid off in a biosafety cabinet for 40 min. Spots were air-dried with the lid on. Plates were inverted and incubated for 48 h at 30°C. Plates were imaged using a flatbed scanner.

### EMS mutagenesis

Overnight cultures of PA14 *tfpY*::FRT were subcultured 1:100 in LB media and grown at 37°C with shaking at 200 rpm. Subcultures were standardized to an OD_600_ of 0.3 in LB media, and 990 µL of each culture was aliquoted into microcentrifuge tubes. Ten microliters of ethyl methanesulfonate (EMS) (Sigma-Aldrich) stock solution was added to achieve a final concentration of 100 mM. Cells were vortexed for 30 s and then incubated at 37°C for 1 h. After incubation, the cells were pelleted at 21,000 × *g* for 1 min and the supernatant was removed. Cells were then resuspended in 1 mL of 1× PBS and pelleted again at 21,000 × *g* for 1 min. This wash step was repeated a second time. The pellet was resuspended in 1 mL of LB media. The entire 1 mL of culture was pipetted in 10-µL spots onto 1.5% LB agar plates, which were air-dried with the lid off in a biosafety cabinet for 30 min. The plates were then incubated for 24 h at 37°C. Motile flares that emerged from these spots were picked using a toothpick and used to stab-inoculate cell culture-treated Nunc OmniTray Single-Well Plates containing 1% LB agar. These plates were then incubated at 37°C overnight. After incubation, the agar was carefully removed and a sterile cotton swab dipped in LB media was used to scrape up cells at the edge of the twitching zones. The swab was then used to streak for single colonies on 1.5% LB agar plates. Twitching motility of single colonies was tested and −80°C glycerol stocks of strains that exhibited greater motility than the *tfpY*::FRT parent strain were made. Genomic DNA from these strains and their parent was extracted and sequenced using Illumina sequencing at SeqCenter (Pittsburgh, PA, USA). SeqCenter also compared the EMS mutants to the parent strain (*tfpY*::FRT) using breseq (41) to identify mutations.

### Figures and statistical analyses

Graphs were generated using GraphPad Prism version 10.2.3. One-way ANOVA statistical analysis was performed, followed by Turkey’s multiple comparisons test on the twitching zone area measurements using GraphPad Prism (*P* < 0.05 considered statistically significant).

## Data Availability

Annotated phage sequences were submitted to GenBank and are available at the following accession numbers: PQ571159 (*Pseudomonas* phage Forsyth), PQ571160 (*Pseudomonas* phage Cline), and PQ571161 (*Pseudomonas* phage Haddon).

## SUPPLEMENTAL MATERIAL

Supplemental material jb.00343-24-s0001.pdf Fig. S1 to S5, Table S1, and Supplemental references.

ASM does not own the copyrights to Supplemental Material that may be linked to, or accessed through, an article. The authors have granted ASM a non-exclusive, world-wide license to publish the Supplemental Material files. Please contact the corresponding author directly for reuse.
