# Supplementary material for: Structural conservation and functional role of TfpY-like proteins in type IV pilus assembly
Source: J Bacteriol. 2025 Jan 16;207(2):e00343-24. doi: 10.1128/jb.00343-24 (PMC11841053; doi:10.1128/jb.00343-24)
Supplement: Supplemental material — Fig. S1 to S5, Table S1, and Supplemental references. [file jb.00343-24-s0001.pdf]

Supplemental Information

Structural conservation and functional role of TfpY-like proteins in type IV pilus assembly

Ikram Qaderi[a,b], Isabelle Chan[a,b], Hanjeong Harvey[a,b], and Lori L. Burrows[a,b#]

[a]Department of Biochemistry and Biomedical Sciences, McMaster University, Hamilton, ON, Canada

[b]Michael G. DeGroote Institute for Infectious Disease Research, McMaster University, Hamilton, ON, Canada

#Corresponding Author:

Dr. Lori L. Burrows

Email: lori.burrows@mcmaster.ca

This document contains:

Figures S1 to S5;

Table S1; and

Supplemental References.

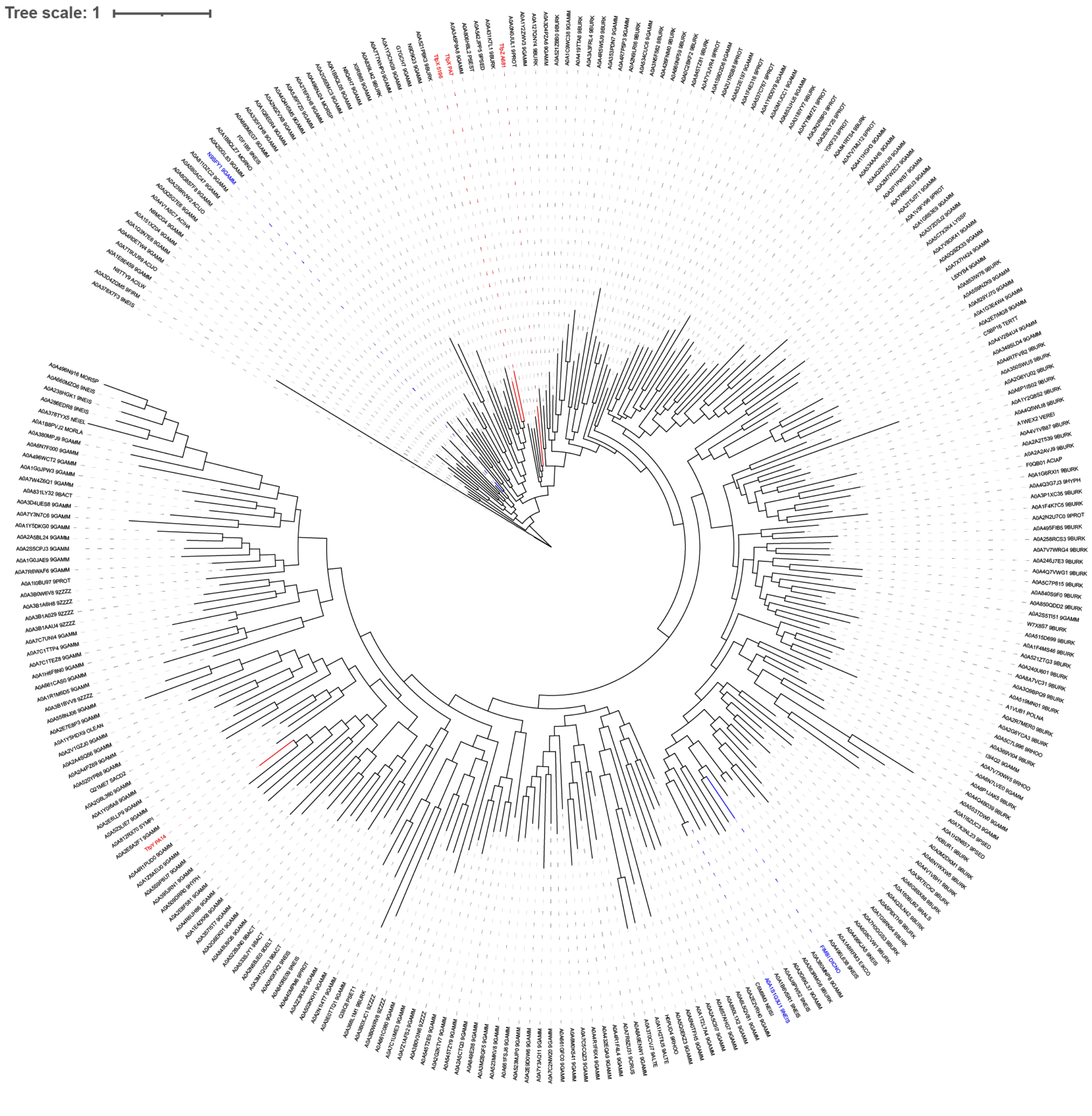

Figure S1. Phylogenetic distribution of TfpY-like proteins. Phylogenetic tree of TfpY-like proteins. Representative *P. aeruginosa* TfpX, TfpY, and TfpY protein sequences used to iteratively search the UniProtKb database using Jackhmmer (1) are shown in red. Representative T4P-expressing species from the phylum *Pseudomonadota* are shown in blue. AlphaFold3 (2) predicted structures for these homologs can be found in **Figure 1**.

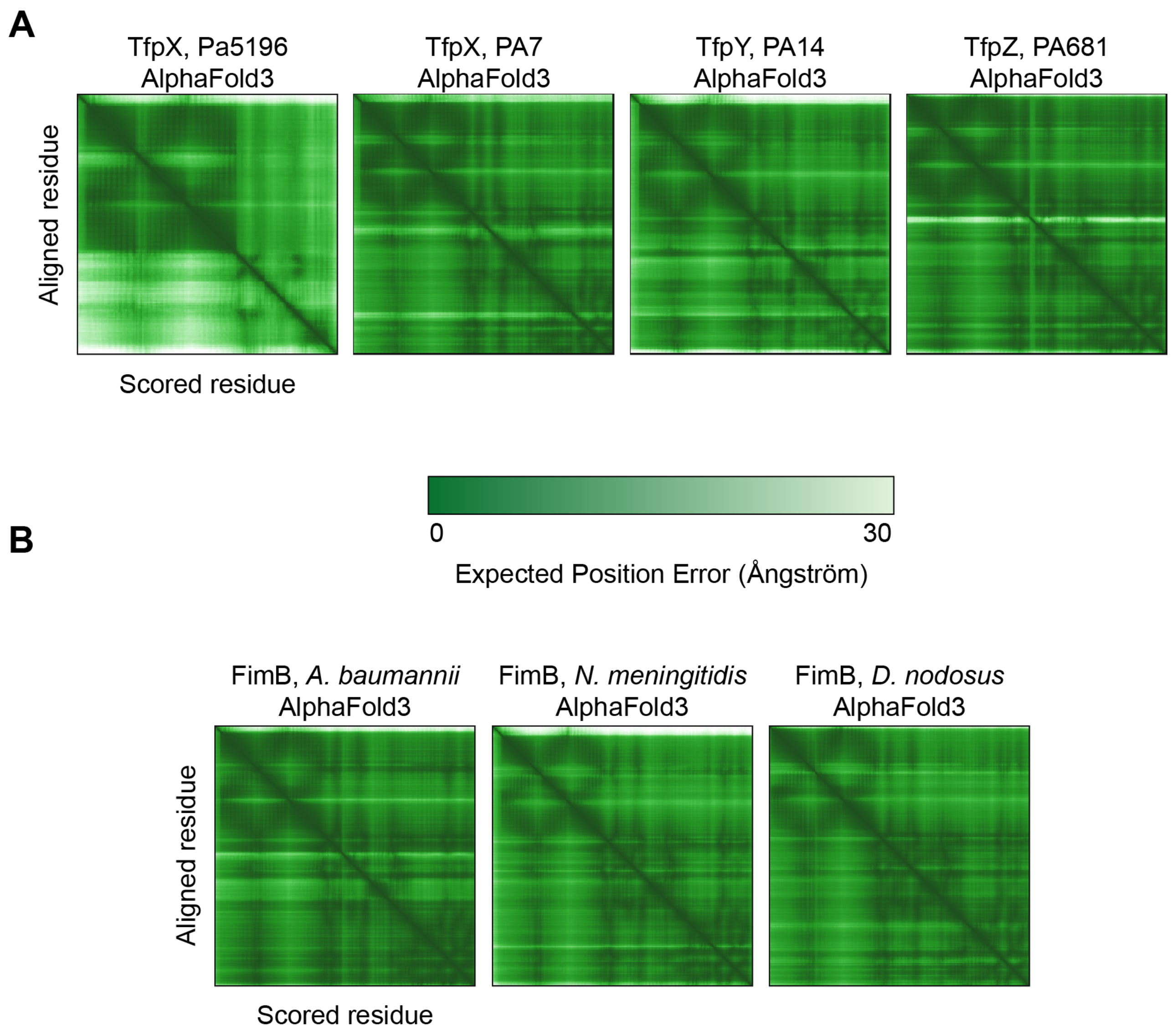

Figure S2. Predicted aligned error plots for AlphaFold3 predicted structures. Error plots for predicted structures in **Figure 1** were generated using AlphaFold3 (2) and then visualized using UCSF ChimeraX (3).

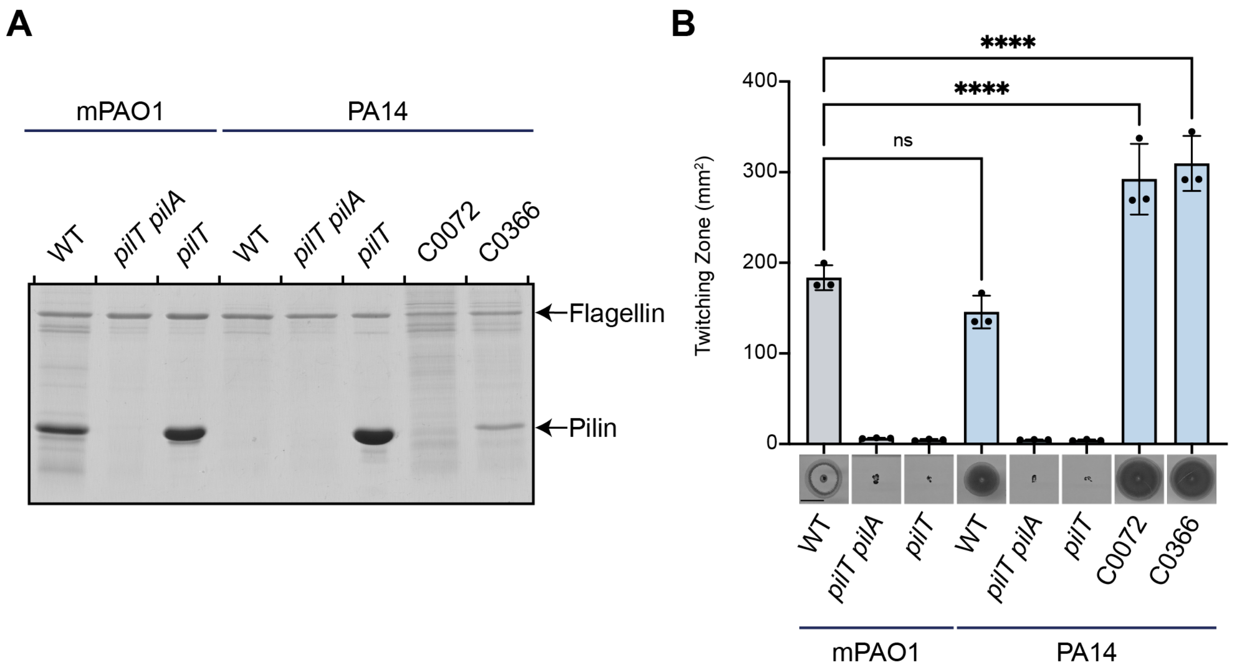

Figure S3. Surface piliation levels do not correlate with twitching motility. A. PA14 and PA14-like strains, WCC C0072 and C0366, produce low levels of recoverable surface pili compared to mPAO1. Coomassie stained SDS-PAGE gel is representative of three independent experiments. **B.** PA14 and PA14-like strains display comparable levels of twitching to mPAO1. Grey bars represent group II strain mPAO1 and blue bars represent group III strains. Twitching images are representative of three independent experiments. Scale bar represents 10 mm. ****$p < 0.0001$.

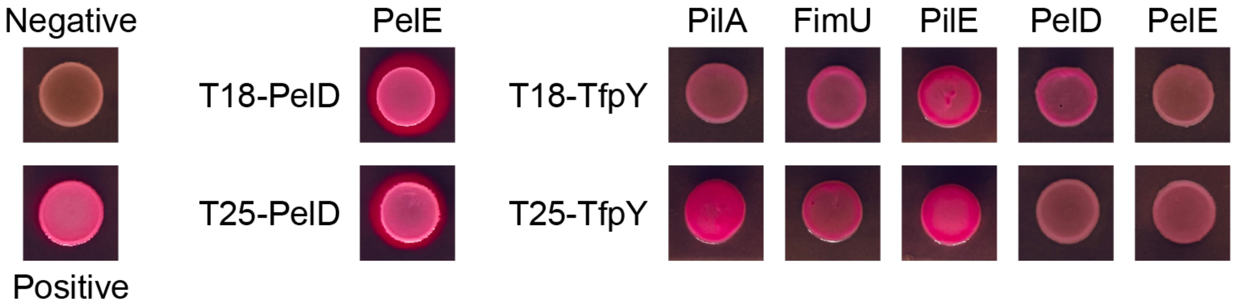

Figure S4. Assessment of non-specific interactions in BACTH assay using, pel polysaccharide biogenesis proteins, PelD and PelE. A strong positive interaction was observed between PelD and PelE, while a weak interaction was detected between T18-TfpY and T25-PelD. No interaction was observed between T25-TfpY and T18-PelD or between TfpY and PelE. Empty pUT18C and pKNT25 plasmids were used as negative controls, while pUT18C-PilS and pKNT25-PilS were used as positive controls. Plates were incubated for 48 h at 30°C. All samples were induced using 0.5 mM IPTG. Images are representative of three independent experiments.

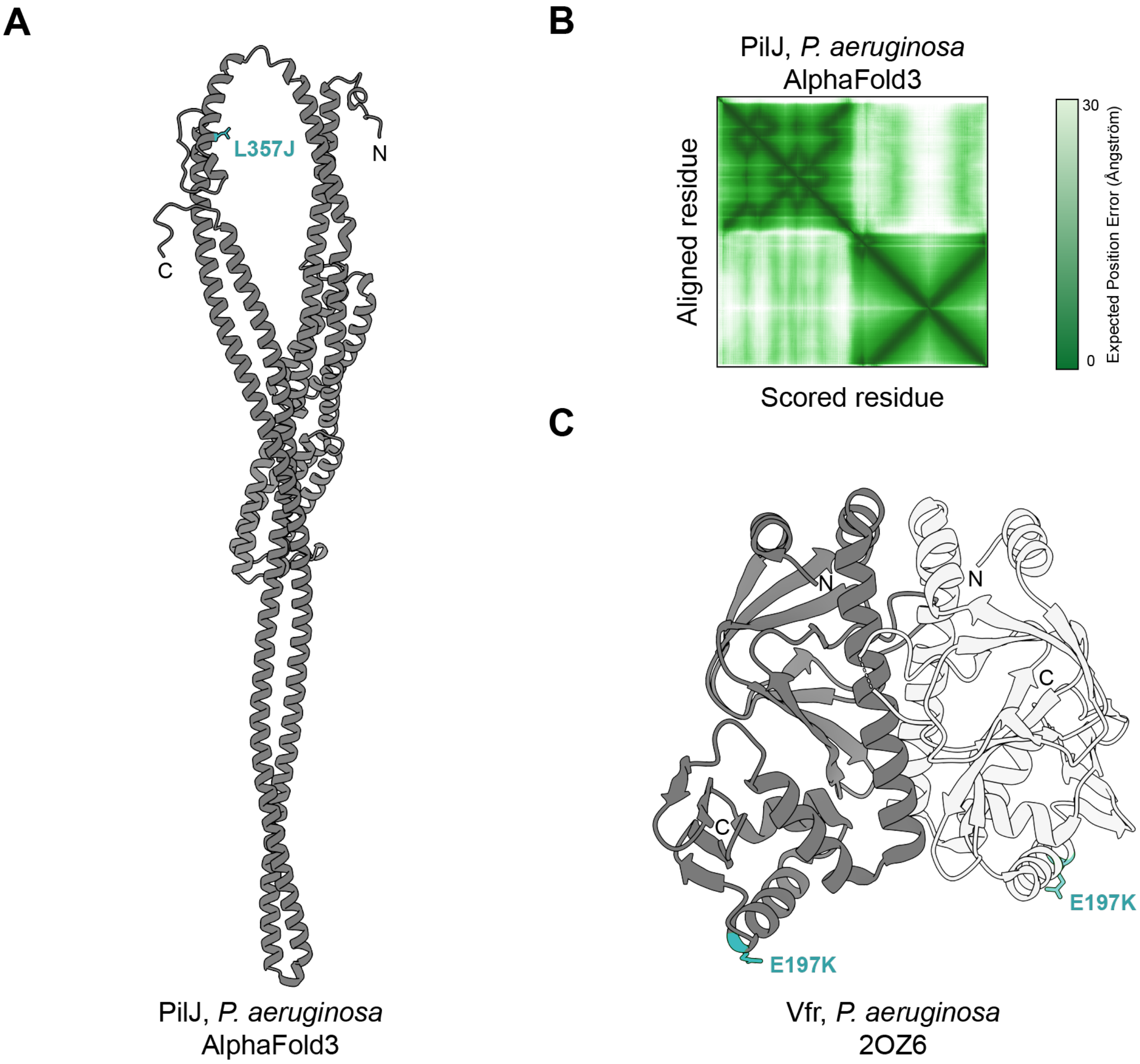

Figure S5. Potential gain-of-function mutations in PilJ and Vfr restore twitching motility in *tfpY* mutants. A. Cartoon model of PilJ in *P. aeruginosa* PA14. **B.** Predicted aligned error plots for the predicted structure of PilJ. **C.** Cartoon model of Vfr dimer (2OZ6). Structure and error plot was predicted using AlphaFold3 (2) and modelled using UCSF ChimeraX (3).

Supplementary Table S1. Plasmids and strains used in this study.

Strain/Plasmid	Characteristics	Source
**Plasmids**		
pEX18Gm	Suicide vector for gene replacement	(4)
pEX18Gm-*pilT*	Deletion construct for PA14 *pilT*	This work
pEX18Gm-*wzy*	Deletion construct for PA14 *wzy*	This work
pEX18Gm-*pilA-tfpY*	Deletion construct for PA14 *pilA-tfpY*	This work
pEX18Ap-$pilA_V$-*tfpZ*::GmFRT	FRT deletion construct for Pa1457 *pilA-tfpZ* cassette	This work
pEX18Gm-$tfpY^{WT}$-V5	Mating construct to introduce C-terminal V5 tag for TfpY	This work
pEX18Gm-$tfpY^{Stop}$	Mating construct to introduce premature stop codon at Leu59 of TfpY	This work
pEX18Gm-$tfpY^{OE}$	Mating construct to introduce cytosines at T2 and T3 of transcriptional terminator U-stretch between PA14 *pilA* and *tfpY*	This work
pEX18Gm-PilJ L357F	Mating construct for PA14 PilJ L357F substitution	This work
pEX18Gm-Vfr E197 K	Mating construct for PA14 Vfr E197K substitution	This work
pEX18Ap-*pilO*::GmFRT	FRT deletion construct for mPAO1 *pilO*	(5)
pBADGr	Arabinose-inducible complementation vector	(6)
pBADGr-*pilA-tfpY*-V5	Complementation construct for PA14 *pilA-tfpY* cassette	This work
pBADGr-$pilA_{IV}$	Complementation construct for Pa5196 *pilA*	(6)
pHERD30T	Arabinose-inducible complementation vector	(7)
pHERD30T-$pilA_{III}$	Complementation construct for PA14 *pilA*	This work
pHERD30T- *pilA-tfpY*-V5	Complementation construct for PA14 *pilA-tfpY* cassette	This work
pHERD30T-$pilA_V$	Complementation construct for Pa1457 *pilA*	(6)

pHERD30T-*pilA*$_V$-*tfpZ*-V5	Complementation construct for Pa1457 *pilA-tfpZ* cassette	This work
pHERD30T-*pilA*$_V$-*tfpY*-V5	Complementation chimeric construct of Pa1457 *pilA* and PA14 *tfpY*	This work
pHERD30T-*cyaB*	Complementation construct for PA14 *cyaB*	This work
pKT25	Vector encoding T25 fragment of *B. pertussis* CyaA	(8)
pKT25-*pilS*	Vector encoding *pilS* fused to T25	(9)
pKT25-*tfpY*	Vector encoding *tfpY* fused to T25	This work
pKT25-*pilA*	Vector encoding *pilA* fused to T25	This work
pKT25-*fimU*	Vector encoding *fimU* fused to T25	This work
pKT25-*pilV*	Vector encoding *pilV* fused to T25	This work
pKT25-*pilW*	Vector encoding *pilW* fused to T25	This work
pKT25-*pilX*	Vector encoding *pilX* fused to T25	This work
pKT25-*pilY1*	Vector encoding *pilY1* fused to T25	This work
pKT25-*pilE*	Vector encoding *pilE* fused to T25	This work
pKT25-*pelD*	Vector encoding *pelD* fused to T25	(10)
pKT25-*pelE*	Vector encoding *pelE* fused to T25	(10)
pUT18C	Vector encoding T18 fragment of *B. pertussis* CyaA	(8)
pUT18C-*pilS*	Vector encoding *pilS* fused to T18	(9)
pUT18C-*tfpY*	Vector encoding *tfpY* fused to T18	This work
pUT18C-*pilA*	Vector encoding *pilA* fused to T18	This work
pUT18C-*fimU*	Vector encoding *fimU* fused to T18	This work
pUT18C-*pilV*	Vector encoding *pilV* fused to T18	This work
pUT18C-*pilW*	Vector encoding *pilW* fused to T18	This work
pUT18C-*pilX*	Vector encoding *pilX* fused to T18	This work
pUT18C-*pilY1*	Vector encoding *pilY1* fused to T18	This work
pUT18C-*pilE*	Vector encoding *pilE* fused to T18	This work
pUT18C-*pelD*	Vector encoding *pelD* fused to T18	(10)
pUT18C-*pelE*	Vector encoding *pelE* fused to T18	(10)
***E. coli* strains**		

DH5α	*F⁻φ80lacZΔM15 Δ(lacZYA-argF)U169 recA1 endA1 hsdR17(rk⁻, mk⁺) phoA supE44 thi-1 gyrA96 relA1 λ−*	Invitrogen
SM10	*thi-1 thr leu tonA lacY supE recA::RP4-2-Tc::Mu (Km^R)*	Invitrogen
BTH101	Bacterial two-hybrid reporter strain	Euromedex
***P. aeruginosa* strains**		
PA14	WT	(11)
PA14 + pBADGr	WT complemented with pBADGr	This work
PA14 + pHERD30T	WT complemented with pHERD30T	This work
PA14 *pilA*	Deletion of *pilA*	(12)
PA14 *pilA* + pHERD30T	Deletion of *pilA* complemented with pHERD30T	This work
PA14 *tfpY*	FRT insertion in *tfpY*	(6)
PA14 *tfpY* + pHERD30T	FRT insertion in *tfpY* complemented with pHERD30T	This work
PA14 *tfpY* + pHERD30T-*cyaB*	FRT insertion in *tfpY* complemented with *cyaB*	This work
PA14 *tfpY* PilJ L357F	PA14 *tfpY*::FRT with PilJ L357F chromosomal mutation	This work
PA14 *tfpY* PilJ L357F + pHERD30T	PA14 *tfpY*::FRT with PilJ L357F chromosomal mutation complemented with pHERD30T	This work
PA14 *tfpY* Vfr E197K	PA14 *tfpY*::FRT with Vfr E197K chromosomal mutation	This work
PA14 *tfpY* Vfr E197K + pHERD30T	PA14 *tfpY*::FRT with Vfr E197K chromosomal mutation complemented with pHERD30T	This work
PA14 *pilT*	Deletion of *pilT*	This work
PA14 *pilT* + pBADGr-$pilA_{IV}$	Deletion of *pilT* complemented with $pilA_{IV}$	This work
PA14 *pilT pilA*	Deletion of *pilT* in PA14 *pilA*	This work

PA14 *pilT pilA* + pBADGr-$pilA_{IV}$	Deletion of *pilT* in PA14 *pilA* complemented with $pilA_{IV}$	This work
PA14 *pilT* $tfpY^{WT}$	Deletion of *pilT* in PA14 *tfpY*-V5	This work
PA14 *pilT* $tfpY^{WT}$ + pBADGr-$pilA_{IV}$	Deletion of *pilT* in PA14 $tfpY^{WT}$ complemented with $pilA_{IV}$	This work
PA14 *pilT* $tfpY^{Stop}$	Deletion of *pilT* in PA14 $tfpY^{Stop}$	This work
PA14 *pilT* $tfpY^{Stop}$ + pBADGr-$pilA_{IV}$	Deletion of *pilT* in PA14 $tfpY^{Stop}$ complemented with $pilA_{IV}$	This work
PA14 *pilT* $tfpY^{OE}$	Deletion of *pilT* in PA14 $tfpY^{OE}$	This work
PA14 *pilT* $tfpY^{OE}$ + pBADGr-$pilA_{IV}$	Deletion of *pilT* in PA14 $tfpY^{OE}$ complemented with $pilA_{IV}$	This work
PA14 *pilT pilY1*	Deletion of *pilT* in PA14 *pilY1*	This work
PA14 *wzy*	Deletion of *wzy*	This work
PA14 EMS Mutant 1	PA14 *tfpY*::FRT mutant exposed to 100 mM of EMS	This work
PA14 EMS Mutant 2	PA14 *tfpY*::FRT mutant exposed to 100 mM of EMS	This work
PA14 *pilA tfpY* + pBADGr	Deletion of *pilA-tfpY* in PA14 complemented with pBADGr	This work
PA14 *pilA tfpY* + pBADGr-*pilA-tfpY*-V5	Deletion of *pilA-tfpY* in PA14 complemented with *pilA-tfpY*-V5	This work
WCC C0072	PA14-like clinical isolate	(13)
WCC C0366	PA14-like clinical isolate	(13)
mPAO1	WT	(14)
mPAO1 *pilT*	ISphoA/hah transposon insertion at position 885 of *pilT*	(14)
mPAO1 *pilT pilA*	ISphoA/hah transposon insertion at position 163 of *pilA* and FRT insertion in the NruI site of *pilT*	(15)
mPAO1 *pilO*	FRT insertion in *pilO*	This work
Pa1457 + pHERD30T	(also known as PA281457)	This work

Pa1457 *pilA tfpZ* + pHERD30T	FRT insertion in *pilA-tfpZ* cassette complemented with pHERD30T	This work
Pa1457 *pilA tfpZ* + pHERD30T-$pilA_{III}$	FRT insertion in *pilA-tfpZ* cassette complemented with $pilA_{III}$	This work
Pa1457 *pilA tfpZ* + pHERD30T-*pilA-tfpY*-V5	FRT insertion in *pilA-tfpZ* cassette complemented with *pilA-tfpY*-V5	This work
Pa1457 *pilA tfpZ* + pHERD30T-$pilA_V$	FRT insertion in *pilA-tfpZ* cassette complemented with $pilA_V$	This work
Pa1457 *pilA tfpZ* + pHERD30T-*pilA-tfpZ*-V5	FRT insertion in *pilA-tfpZ* cassette complemented with *pilA-tfpZ*-V5	This work
Pa1457 *pilA tfpZ* + pHERD30T-$pilA_V$-*tfpY*-V5	FRT insertion in *pilA-tfpZ* cassette complemented with $pilA_V$-*tfpY*-V5	This work
**Bacteriophage**		
P2B9	DsDNA LPS-targeting bacteriophage isolated from sewage	(16)
DMS3	DsDNA pilus-targeting bacteriophage	(17)
Haddon	DsDNA pilus-targeting bacteriophage	This work
Cline	DsDNA pilus-targeting bacteriophage	This work
Forsyth	DsDNA pilus-targeting bacteriophage isolated from sewage	This work

Supplementary Table S2. Primers used in this study.

**Chromosomal Deletion Primers**			
**Gene**	**Fragment**	**Forward (5’ → 3’)**	**Reverse (5’ → 3’)**
pilT	Upstream	TTTTCCCGGGGATTCCTTCAAGAAGCCGGCG	CGAGGGTCTGCATGGTTGATCCGGCGTACATCGC

	Downstream	TACGCCGGATCAACCATGCAGACCCTCGACATGTG	TTTTAAGCTTGGAAGAAGTGGATGATCCGCTC
pilA-tfpY	Upstream	TTTTGAATTCGGTAAATCTTTTCATAGGGCTCG	TCAATATCCAGACGGGTGTAATCCTGATATGCCGG
	Downstream	ATCAGGATTACACCCGTCTGGATATTGAAAGCGC	TTTTGGATCCGCTGTTCCAGCTAAAAGGTCC
pilA-tfpZ	FRT insertion	AGATGAATTCATGAAAGCTCAAAAAGGCTT	AGATAAGCTTACGAATGAGCTGCTCTACCG
wzy	Upstream	ACAGAATTCGGCGTTCAAGTAATAGTCGATGC	GAGTAAGCCAGGTCAGCCCATACACTAAAAATCTTCCGTGC
	Downstream	AGATTTTTAGTGTATGGGCTGACCTGGCTTACTCTGC	ACAGGATCCCCTTGCTACACACGTAGTCCC
**Chromosomal Knock-in Primers**			
PilJ L357F		TTTTGAATTCCATGGAAATCGTAATCGTGGTCG	TTTTGGATCCCTATGCTCAGGCCTGCTCC
Vfr E197K		TTTTGAATTCGATCAGGACCATCTCCATAGGG	TTTTGGATCCCGGTTCTGCAGAAGATCCTCG
tfpY-V5	Upstream	TTTGAATTCGAGAGATACATGAAAGCT	CTATTTCGAACAAAGCCCCGGGATACC
	Downstream	GGTATCCCCGGGGCTTTGTTC	AAAAGGATCCAAAACCACATCGC
$tfpY^{Stop}$	Upstream	TTTGAATTCGAGAGATACATGAAAGCT	CAATGGCCCCAAAACTTAGTCGACAGC

	Downstream	GCCGCTGTCGACTAAGTTTTGGGGCCA	TTTAAGCTTTCAGGTGCTGTCCAGGCCC
tfpY^{OE}	Upstream	TTTGAATTCGAGAGATACATGAAAGCT	GTACTATAACCACTCTCCCACATAAAAGGAGCCCCTCTTAG
	Downstream	CTAAGAGGGGCTCCTTTTATGTGGGAGAGTGGTTATAGTAC	AAAAGGATCCAAAACCACATCGC
**Complementation Primers**			
pilA$_{III}$		TTTGAATTCGAGAGATACATGAAAGCT	TTTTAAGCTTTTAGCGGCATTCGCTCGGAG
pilA-tfpY-V5		TTTGAATTCGAGAGATACATGAAAGCT	TTTAAGCTTTCAGGTGCTGTCCAGGCCC
pilA-tfpZ-V5		TTTTGAATTCATGAAAGCTCAAAAAGGCTTCACTTTG	TTTTAAGCTTTTACGTAGAATCGAGACCGAGGAGAGGGTTAGGGATAGGCTTACCGCCTTGCTTCCAGGCCC
pilA$_{V}$-*tfpY*	Upstream	TTTTGAATTCATGAAAGCTCAAAAAGGCTTCACTTTG	TCAGCATTAGCCTATTAGCGGCACTGAGCAGGA
	Downstream	CTCAGTGCCGCTAATAGGCTAATGCTGAAAAGAGCCC	TTTTAAGCTTTCACGTAGAATCGAGACCGAGG
cyaB		TTTTGAATTCATGAAGCCTACCCTCCCCGA	TTTTAAGCTTTCAGAGGATGACCTTGTCGCG
tfpY	T18	TTTTGGATCCAGTGGTTATAGTGCATTTCGCTG	TTTTGAATTCCCAGTCGCAGCATCACTCTT
	T25		
pilA	T18		

	T25	TTTTCTAGAGTTTACCTTGATCGAACTGATGATCG	TTTTCCCGGGATTAGCGGCATTCGCTCGG
fimU	T18	TTTTTCTAGAGTTTTCCCTGATCGAGTTGATGATG	TTTTCCCGGGCTCAGTTACAGCTGTCCGGT
	T25		
pilV	T18	TTTTTCTAGAGTTCAGCATGATCGAAGTACTGGTT	TTTTGAATTCGATTCGGAGCGGACGCTG
	T25		TTTTGAATTCTTTCGGAGCGGACGCTG
pilW	T18	TTTTTCTAGAGCTATCGATGGTAGAACTGCTCGT	TTTTCCCGGGGTCATGGCATGAGATTCCTGATG
	T25		
pilX	T18	TTTTTCTAGAGTCCACGTTGTTGATCTCGCTG	TTTTCCCGGGGTCAGTTGGTATACAGGCGTGC
	T25		
pilY1	T18	TTTTCCCGGGGATGATCCACCAGATTACCCGC	TTTTGAATTCGATCATTTCTCCTCGACGACCC
	T25		TTTTGAATTCTTCATTTCTCCTCGACGACCC
pilE	T18	TTTTTCTAGAGTTCACTCTCATCGAGTTGATGATCG	TTTTCCCGGGTTTAGCGCCAGCATTCTGCA
	T25		

SUPPLEMENTARY REFERENCES

1. Potter SC, Luciani A, Eddy SR, Park Y, Lopez R, Finn RD. 2018. HMMER web server: 2018 update. Nucleic Acids Res 46:W200–W204.

2. Abramson J, Adler J, Dunger J, Evans R, Green T, Pritzel A, Ronneberger O, Willmore L, Ballard AJ, Bambrick J, Bodenstein SW, Evans DA, Hung C-C, O'Neill M, Reiman D, Tunyasuvunakool K, Wu Z, Žemgulytė A, Arvaniti E, Beattie C, Bertolli O, Bridgland A, Cherepanov A, Congreve M, Cowen-Rivers AI, Cowie A, Figurnov M, Fuchs FB, Gladman H, Jain R, Khan YA, Low CMR, Perlin K, Potapenko A, Savy P, Singh S, Stecula A, Thillaisundaram A, Tong C, Yakneen S, Zhong ED, Zielinski M, Žídek A, Bapst V, Kohli P, Jaderberg M, Hassabis D, Jumper JM. 2024. Accurate structure prediction of biomolecular interactions with AlphaFold 3. Nature 630:493–500.

3. Meng EC, Goddard TD, Pettersen EF, Couch GS, Pearson ZJ, Morris JH, Ferrin TE. 2023. UCSF ChimeraX: Tools for structure building and analysis. Protein Sci 32:e4792.

4. Hoang TT, Karkhoff-Schweizer RR, Kutchma AJ, Schweizer HP. 1998. A broad-host-range Flp-*FRT* recombination system for site-specific excision of chromosomally-located DNA sequences: application for isolation of unmarked *Pseudomonas aeruginosa* mutants. Gene 212:77–86.

5. Ayers M, Sampaleanu LM, Tammam S, Koo J, Harvey H, Howell PL, Burrows LL. 2009. PilM/N/O/P Proteins Form an Inner Membrane Complex That Affects the Stability of the *Pseudomonas aeruginosa* Type IV Pilus Secretin. J Mol Biol 394:128–142.

6. Asikyan ML, Kus JV, Burrows LL. 2008. Novel Proteins That Modulate Type IV Pilus Retraction Dynamics in *Pseudomonas aeruginosa*. J Bacteriol 190:7022–7034.

7. Qiu D, Damron FH, Mima T, Schweizer HP, Yu HD. 2008. PBAD-Based Shuttle Vectors for Functional Analysis of Toxic and Highly Regulated Genes in *Pseudomonas* and *Burkholderia* spp. and Other Bacteria. Appl Environ Microbiol 74:7422–7426.

8. Karimova G, Pidoux J, Ullmann A, Ladant D. 1998. A bacterial two-hybrid system based on a reconstituted signal transduction pathway. Proc Natl Acad Sci 95:5752–5756.

9. Kilmury SLN, Burrows LL. 2016. Type IV Pilins Regulate Their Own Expression via Direct Intramembrane Interactions with the Sensor Kinase PilS. Proc Natl Acad Sci 113:6017–6022.

10. Whitfield GB, Marmont LS, Ostaszewski A, Rich JD, Whitney JC, Parsek MR, Harrison JJ, Howell PL. 2020. Pel Polysaccharide Biosynthesis Requires an Inner Membrane Complex Comprised of PelD, PelE, PelF, and PelG. J Bacteriol 202:10.1128/jb.00684-19.

11. Rahme LG, Stevens EJ, Wolfort SF, Shao J, Tompkins RG, Ausubel FM. 1995. Common Virulence Factors for Bacterial Pathogenicity in Plants and Animals. Science 268:1899–1902.

12. Nguyen Y, Boulton S, McNicholl ET, Akimoto M, Harvey H, Aidoo F, Melacini G, Burrows LL. 2018. A Highly Dynamic Loop of the *Pseudomonas aeruginosa* PA14 Type IV Pilin Is Essential for Pilus Assembly. ACS Infect Dis 4:936–943.

13. Ranieri MRM, Chan DCK, Yaeger LN, Rudolph M, Karabelas-Pittman S, Abdo H, Chee J, Harvey H, Nguyen U, Burrows LL. 2019. Thiostrepton Hijacks Pyoverdine Receptors To Inhibit Growth of *Pseudomonas aeruginosa*. Antimicrob Agents Chemother 63:10.1128/aac.00472-19.

14. Jacobs MA, Alwood A, Thaipisuttikul I, Spencer D, Haugen E, Ernst S, Will O, Kaul R, Raymond C, Levy R, Chun-Rong L, Guenthner D, Bovee D, Olson MV, Manoil C. 2003. Comprehensive transposon mutant library of *Pseudomonas aeruginosa*. Proc Natl Acad Sci 100:14339–14344.

15. Harvey Hanjeong, Habash Marc, Aidoo Francisca, Burrows Lori L. 2009. Single-Residue Changes in the C-Terminal Disulfide-Bonded Loop of the *Pseudomonas aeruginosa* Type IV Pilin Influence Pilus Assembly and Twitching Motility. J Bacteriol 191:6513–6524.

16. Chan DCK, Dykema K, Fatima M, Harvey H, Qaderi I, Burrows LL. 2023. Nutrient Limitation Sensitizes *Pseudomonas aeruginosa* to Vancomycin. ACS Infect Dis 9:1408–1423.

17. Budzik JM, Rosche WA, Rietsch A, O'Toole GA. 2004. Isolation and Characterization of a Generalized Transducing Phage for *Pseudomonas aeruginosa* Strains PAO1 and PA14. J Bacteriol 186:3270–3273.
